# Functional characterization of SHC-like triterpene cyclase genes in azole response and virulence-related traits of *Aspergillus fumigatus*

**DOI:** 10.1080/21505594.2026.2715234

**Published:** 2026-09-05

**Authors:** Kerui Ye, Fangyan Chen, Lingxue Hu, Jinghui Han, Dingchen Li, Mandong Hu, Changjun Wang, Jumei Zeng, Li Han

**Affiliations:** a West China School of Public Health and West China Fourth Hospital, Sichuan University, Chengdu, China; b Department for Disinfection and Infection Control, Chinese PLA Center for Disease Control and Prevention, Beijing, China; c School of Public Health, China Medical University, Shenyang, Liaoning, China; d Department of Infectious Disease Prevention and Control, Jining Center for Disease Control and Prevention, Jining, Shandong, China

**Keywords:** Aspergillus fumigatus, SHC-like triterpene cyclase, squalene, azole susceptibility, transcriptomic remodeling, virulence-related traits

## Abstract

*Aspergillus fumigatus* is a major opportunistic fungal pathogen, and increasing azole resistance poses a challenge for aspergillosis treatment. Squalene is an upstream precursor of ergosterol biosynthesis and may also be utilized by SHC-like triterpene cyclases, suggesting a potential link between squalene-associated metabolism, membrane adaptation, and azole response. However, the roles of SHC-like triterpene cyclase genes in *A. fumigatus* remain unclear. Here, we characterized three candidates, *shc1*, *shc2*, and *shc3*, using comparative bioinformatic analysis, gene deletion, phenotypic assays, azole susceptibility testing, transcriptomics, and host-interaction models. Sequence, genomic-context, phylogenetic, and structural analyses suggested divergence among the three candidates. Individual *shc* deletion caused limited effects on vegetative growth, whereas loss of *shc1* mildly reduced susceptibility to voriconazole and posaconazole, as reflected by twofold MIC increases and lower inhibition rates. Transcriptomic analysis revealed distinct remodeling patterns, with *Δshc3* showing the broadest transcriptional changes despite no detectable MIC shift. Targeted metabolite profiling and PI uptake analysis further supported an association between *shc* deletion, sterol/hopane-type triterpenoid balance, and membrane-associated properties. *shc* deletion also altered epithelial cell interaction phenotypes, while Δ*shc1* showed reduced lethality in *Galleria mellonella*. In clinical isolates, elevated *shc* transcription was associated with azole-resistant backgrounds. These findings suggest functional diversification among SHC-like triterpene cyclase genes and indicate that *shc1* may contribute to azole-associated adaptation and virulence-related traits in *A. fumigatus*.

## Introduction

*Aspergillus fumigatus* is a filamentous saprophytic fungus that is extensively distributed in various environmental contexts. Conidial concentrations in the air generally range from 0.2 to 15 conidia per cubic meter [1,2]. The conidia, around 2–3 µm in diameter, exhibit remarkable capacity for airborne dispersal. The surface of *A. fumigatus* conidia possesses a hydrophobic protein layer and melanin pigment that successfully avoid detection by the host immune system. Secreted virulence factors, including gliotoxin, can harm pulmonary vascular endothelium and facilitate vascular invasion [3–5]. The characteristics of *A. fumigatus* indicate that inhalation of its spores generally results in mild allergic reactions in immunocompetent individuals; however, it can cause severe invasive aspergillosis (IA) in immunocompromised populations. In recent years, the intensification of cancer chemotherapy regimens, the widespread adoption of organ transplantation techniques, and the extensive use of immunomodulatory drugs have contributed to an annual increase in the number of immunocompromised individuals [6]. The global burden of IA has increased, with approximately 250,000 new cases reported annually worldwide. The 30-day mortality rate of IA ranges from 52% to 78%, with rates exceeding 90% in patients with hematologic malignancies undergoing hematopoietic stem cell transplantation [7,8]. Voriconazole and isavuconazole are recommended first-line options for invasive aspergillosis, whereas posaconazole is commonly used for prophylaxis, salvage therapy, or selected clinical settings, and itraconazole is more often used in other forms of aspergillosis rather than as first-line therapy for IA [9]. Azoles primarily target the 14-α-demethylase encoded by cyp51A, thereby disrupting ergosterol biosynthesis and fungal membrane function [10,11]. The global resistance rate of *A. fumigatus* to azole drugs is exhibiting a consistent upward trend, influenced by two primary factors: the irrational application of agricultural azole fungicides, which results in the selective enrichment of azole-resistant *A. fumigatus* in the environment, and the induction of adaptive resistance in strains as a consequence of extended clinical azole therapy [12–14]. Among the best-characterized mechanisms, *cyp51A* alterations play a central role in azole resistance. Tandem repeat (TR) mutations, including TR34 and TR46, are promoter duplications that increase *cyp51A* expression, while accompanying coding mutations, typically L98H or Y121F/T289A, alter the azole target enzyme. Together, these changes reduce azole susceptibility [15]. However, azole susceptibility is not determined solely by *cyp51A*. Increasing evidence suggests that non-*cyp51A* mechanisms, including transcriptional regulation, drug efflux, stress-response pathways, mitochondrial function, and membrane lipid remodeling, can also contribute to azole adaptation [16]. Therefore, it is essential to investigate new mechanisms that regulate resistance in *A. fumigatus* and to identify potential targets for intervention.

Squalene serves as an essential precursor in the biosynthesis pathway of ergosterol. Squalene epoxidase encoded by *erg1* catalyzes the oxidation of squalene to yield 2,3-epoxysqualene, which subsequently undergoes a series of enzymatic reactions leading to the formation of ergosterol [17]. This pathway is located upstream of the target site for azole drugs. Squalene-hopene cyclase (SHC) is a triterpene cyclase that primarily catalyzes the synthesis of hopene derivatives from squalene [18], utilizing the same substrate pool as squalene epoxidase. Initially isolated from *Alicyclobacillus acidocaldarius* [19], SHC facilitates the synthesis of hopanoids, essential elements of prokaryotic cell membranes that modulate membrane fluidity and permeability [20], similar to the role of sterols in eukaryotic membranes [21].

SHCs and oxidosqualene cyclases (OSCs) belong to the broader triterpene cyclase superfamily. Bacterial SHCs catalyze the cyclization of squalene to hopene-type triterpenoids, whereas fungal OSC/ERG7-like enzymes generally catalyze oxidosqualene cyclization in sterol or triterpenoid metabolism. Because fungal triterpene cyclase-like proteins can share sequence features with both SHC-like and OSC-like enzymes, their precise enzymatic products cannot be inferred from sequence annotation alone. Therefore, in this study, we refer to the analyzed genes as SHC-like triterpene cyclase genes.

SHC-like proteins have also been reported in fungi. In *Schizosaccharomyces japonicus*, SHC-derived hopanoids can partially substitute for sterols under anaerobic conditions, thereby preserving membrane integrity and supporting survival in oxygen-limited environments [22,23]. In *A. fumigatus*, *shc1* has been implicated in fumihopaside-type secondary metabolism, including hopane-type triterpenoid-related products, suggesting that SHC-like triterpene cyclases may participate in fungal secondary metabolism [24]. Recent studies on SHC have focused on its structure, catalytic mechanism, and industrial applications [25–29]; however, the physiological function of SHC in pathogenic fungi, as well as its association with environmental adaptability and antifungal drug resistance, is still limited. Our previous studies on the metabolic enzyme phospholipid phosphatase *sac1* revealed that sac1-deficient strain showed significantly reduced expression of the SHC-encoding gene (AFUA_5G00110) in comparison to parental strains. This observation indicates a possible link between SHC and sac1-mediated regulation of cell membrane function, which may influence the growth, drug resistance, or virulence of *A. fumigatus* [30].

Here, we systematically characterized three SHC-like triterpene cyclase genes, *shc1*, *shc2*, and *shc3*, in *A. fumigatus*. By combining gene deletion, phenotypic assays, azole susceptibility testing, transcriptomic profiling, and infection models, we investigated whether these candidates contribute to azole response, metabolic adaptation, and virulence-related traits.

## Materials and methods

### Strains and culture conditions

The *A. fumigatus* strains used in this study are listed in Table 1. The nonhomologous end-joining-deficient *A. fumigatus* strain *CEA17Δku80*, a generous gift from Jean Paul Latgé, was used as the parental strain for the construction of all deletion mutants [31]. Af293 AFUA locus tags were used throughout the manuscript for consistency with widely used public annotations. Related A1163/AFUB orthologs of *shc1*, *shc2*, and *shc3* are listed in Table S4. *A. fumigatus* strains were cultured on Aspergillus minimal medium (AMM) plates at 37°C for 3–5 days to obtain conidia. Conidia were harvested with phosphate-buffered saline containing 0.1% Tween 20 (PBST), filtered through a 40-µm membrane to remove hyphal fragments, counted using a hemocytometer, and adjusted to a final concentration of 1 × 10^8^ conidia/mL. Key reagents and materials used in this study are listed in Table S1.

**Table 1.** Genetic background and MIC values of azole drugs for *A. fumigatus Δshc1, Δshc2*, and *Δshc3* mutants.

Strains	Genetic background	MIC (µg/mL)			Reference
		ITC	VOR	POS	
*Δku80*	*Δku80*	≥1	0.5	0.25	[31]
*Δshc1*	*Δku80Δshc1*::hph	≥1	1	0.5	This work
*Δshc2*	*Δku80Δshc2*::hph	≥1	0.5	0.25	This work
*Δshc3*	*Δku80Δshc3*::hph	≥1	0.5	0.25	This work

### DNA and RNA extraction, and cDNA preparation

A 10-µL aliquot of *A. fumigatus* conidial suspension (approximately 1 × 10^8^ conidia/mL) was inoculated into liquid minimal medium (MM) and incubated at 37°C with shaking at 200 rpm for 24 h. Mycelial pellets were collected by filtration through sterile gauze, frozen in liquid nitrogen, and ground into a fine powder for subsequent nucleic acid extraction. Genomic DNA was extracted using a fungal genomic DNA extraction kit (Hangzhou Bioer Technology Co., Ltd., Cat. No. BSC14M1) according to the manufacturer’s instructions. Total RNA was isolated using TransZol Up Plus RNA Kit (TransGen Biotech Co., Ltd., Cat. No. ER501-01-V2). First-strand cDNA was synthesized using TransScript All-in-one First-Strand cDNA Synthesis SuperMix for qPCR (TransGen Biotech Co., Ltd., Cat. No. AT341) with an anchored oligo(dT)_18_ primer. The resulting cDNA was diluted and used as a template for subsequent qPCR analysis.

### Construction of mutant strains

The primer sequences used in this study are listed in Table S2. Homologous arms of approximately 1.0–2.0 kb flanking each target gene were amplified from *CEA17Δku80* genomic DNA. Gene deletion cassettes containing the 5' flanking region, the hygromycin resistance cassette (*hph*), and the 3' flanking region were constructed by fusion PCR as described previously [32]. The purified deletion cassettes were introduced into *CEA17Δku80* protoplasts by PEG-mediated transformation. Transformants were selected on AMM plates containing hygromycin B (200 µg/mL; Beijing Coolaber Technology Co., Ltd., Cat. No. CH6361) and purified by two rounds of subculturing under hygromycin selection.

Putative deletion mutants were screened by diagnostic PCR. Correct integration of the deletion cassette was verified by 5'- and 3'-junction PCR, and loss of the target open reading frame was examined by target-gene PCR. Several putative *Δshc1* transformants were screened by diagnostic PCR, and transformant 4, showing the expected target-gene loss and 5'/3' junction amplification, was selected as the verified *Δshc1* mutant for subsequent analyses. The *Δshc2* and *Δshc3* mutants were verified by the same diagnostic PCR strategy (Fig. S1). One fully verified representative mutant for each gene deletion was selected for subsequent phenotypic, transcriptomic, and infection-model analyses [33].

### Identification and bioinformatic analysis of SHC-like triterpene cyclase genes

To identify candidate SHC-like triterpene cyclase genes in *A. fumigatus*, the protein sequence of the well-characterized squalene-hopene cyclase from *A. acidocaldarius* was used as the query for BLASTp analysis against the predicted proteome of *A. fumigatus Af293* in the NCBI database. Query coverage, percent identity, E-value, protein accession, and functional annotation were recorded for the main hits. The top-ranked candidate genes and representative OSC-like triterpene cyclase paralogs were included in subsequent comparative analyses. To evaluate secondary metabolite gene-cluster contexts, the *A. fumigatus* Af293 genome was analyzed using antiSMASH version 8.0.4. Predicted cluster regions, cluster types, similarity to known MIBiG biosynthetic gene clusters, and similarity scores or confidence levels were recorded for *shc1*, *shc2*, and *shc3*.

Multiple sequence alignments were conducted between SHC1–3, the SHC sequences from *A. acidocaldarius* and representative OSC-like comparator proteins to examine conserved regions and catalytic motifs. Phylogenetic analysis was performed using the maximum likelihood method in MEGA 11, with branch support evaluated using 1,000 bootstrap replicates.

Three-dimensional structures of SHC1, SHC2, and SHC3 were predicted using AlphaFold3. The predicted structures were visualized and superimposed with the resolved *A. acidocaldarius* SHC structure using PyMOL, and RMSD values were calculated to estimate structural similarity.

### Colony growth, conidia formation, and germination capacity assay

To assess the effect of *shc* deletion on colony growth, 1 × 10^5^ conidia were inoculated at the center of Aspergillus minimal medium (AMM) plates and incubated at 28°C, 37°C, and 48°C. Colony diameters were measured and documented by photography at the indicated time points. Conidiation was assessed by culturing each strain on AMM plates for 72 h. Conidia were harvested with phosphate-buffered saline containing 0.1% Tween 20 (PBST) and quantified using a hemocytometer. Results were expressed as conidia per plate or conidia per milliliter.

For liquid growth monitoring, 1 × 10^5^ conidia were inoculated into RPMI-1640 medium (Thermo Fisher Scientific, Inc., Cat. No. 31,800,022) supplemented with 2% glucose and 165 mM MOPS buffer (Beijing Biotopped Technology Co., Ltd., Cat. No. M6420T) in 24-well plates. Cultures were incubated at 37°C, and OD_600_ values were recorded at defined intervals using a microplate reader. Endpoint OD_600_ values at 30 h were used for statistical comparison [34,35].

### Antifungal susceptibility and inhibition-rate assays

Antifungal susceptibility testing was performed according to the EUCAST definitive document E.Def 9.3 for filamentous fungi. Voriconazole (TCI, Cat. No. V0116), itraconazole (Beijing Solarbio Science & Technology Co., Ltd., Cat. No. I8250), and posaconazole (Anhui Senrise Technologies Co., Ltd., Cat. No. E1200070050) were prepared in a twofold serial dilution in double-strength RPMI-1640 medium (Thermo Fisher Scientific, Inc., Cat. No. 31,800,022) supplemented with 2% glucose and buffered with MOPS. Drug dilutions were dispensed into 96-well flat-bottom microplates, with growth control and sterility control wells included in parallel. Fresh conidia were harvested from strains grown on solid medium, suspended in sterile water containing 0.1% Tween 20, and counted using a hemocytometer. The inoculum was adjusted according to EUCAST recommendations to obtain a final concentration of 1 × 10^5^ −2.5 × 10^5^ CFU/mL per well. Microplates were incubated statically at 37°C, and MIC values were determined after 48 h by visual inspection. The MIC was defined as the lowest antifungal concentration that completely inhibited visible fungal growth [36,37].

For subinhibitory azole assays, strains were grown on AMM plates containing 0.125 µg/mL POS or 0.25 µg/mL VOR. Colony diameters were measured at the same incubation time point under azole-treated and drug-free conditions. For each strain, the drug-free condition of the same strain was used as its corresponding control. The inhibition rate was calculated as follows: inhibition rate (%) = [1 − (*D*_drug_/*D*_control_)] × 100, where *D*_drug_ represents the colony diameter under azole treatment and *D*_control_ represents the colony diameter of the corresponding drug-free control.

### Transcriptomics analysis

For RNA-seq analysis, *CEA17Δku80*, Δ*shc1*, Δ*shc2*, and Δ*shc3* were analyzed under basal, drug-free conditions. Three independent biological replicates were prepared for each strain. Conidia were inoculated into 100 mL of liquid minimal medium (MM) at a final concentration of 1 × 10^6^ conidia/mL and cultured at 37°C with shaking at 200 rpm for 24 h. Mycelia were collected by filtration through a 40 µm cell strainer, immediately frozen in liquid nitrogen, and ground into powder for total RNA extraction.

RNA quality assessment, library construction, sequencing, and primary bioinformatic processing were performed by Novogene Biotechnology Co., Ltd. RNA integrity was assessed using an Agilent 2100 Bioanalyzer. mRNA was enriched from total RNA using oligo(dT) magnetic beads, fragmented, reverse-transcribed into cDNA, end-repaired, adapter-ligated, PCR-amplified, and purified to generate standard mRNA-seq libraries. Libraries were sequenced on an Illumina platform using paired-end 150-bp sequencing.

Raw reads were filtered using fastp to remove adapter-containing reads, reads containing undetermined bases, and low-quality reads. Clean reads were mapped to the *A. fumigatus* Af293 reference genome (GCF_000002655.1/ASM265v1) using HISAT2. Gene-level read counts were generated using featureCounts, and expression levels were calculated as FPKM values. Differential expression analysis was performed using DESeq2 with *CEA17Δku80* as the reference group for each deletion mutant. *p* values were adjusted using the Benjamini–Hochberg method. Genes with an adjusted *p* value < 0.05 and |log_2_(fold change)| ≥1 were defined as differentially expressed genes. Principal component analysis, hierarchical clustering, volcano plots, Venn diagrams, and KEGG enrichment analysis were performed using R-based pipelines. KEGG enrichment analysis was performed using clusterProfiler.

### RT-qPCR assay

Real-time quantitative PCR (RT-qPCR) was performed to determine the transcription levels of target genes. Reverse-transcribed cDNA (see DNA and RNA extraction, and cDNA preparation) was diluted and used as a template for amplification on a Roche LightCycler real-time PCR system. RT-qPCR reactions were carried out using TransStart Top Green qPCR SuperMix (TransGen Biotech Co., Ltd., Cat. No. AQ131) as described previously [30]. A melting curve analysis was performed to verify amplification specificity. Each sample was analyzed with three technical replicates and at least three independent biological replicates. Relative transcript levels were calculated using the 2^−^ΔΔCt^ method with *tub1* as the reference gene [38,39]. For temperature-response assays, the 37°C group of each gene was used as its calibrator. For azole-treatment assays, the corresponding untreated control was used as the calibrator.

### Cell infection and fluorescence microscopy observation

Human lung adenocarcinoma epithelial A549 cells were purchased from Procell Life Science & Technology Co., Ltd. (Wuhan, China) and used as an alveolar epithelial cell model. Cells were cultured in F-12K medium (Beijing Solarbio Science & Technology Co., Ltd., Cat. No. LA1320) supplemented with 10% fetal bovine serum at 37°C in a humidified atmosphere containing 5% CO_2_. For adhesion assays, A549 cells were seeded onto 24-well plates containing glass coverslips and grown to confluence. Conidia were labeled with fluorescein isothiocyanate (FITC; Beijing Solarbio Science & Technology Co., Ltd., Cat. No. CA1620) and added to the cell monolayers at a multiplicity of infection (MOI) of 10. After incubation at 37°C with 5% CO_2_ for 6 h [40], the supernatant was removed, and cells were treated with nystatin (20 µg/mL; Beijing Solarbio Science & Technology Co., Ltd., Cat. No. N8040) for 4 h to eliminate extracellular conidia. Cells were then gently washed with phosphate-buffered saline (PBS) to remove unattached conidia and fixed with 4% paraformaldehyde (Beijing Solarbio Science & Technology Co., Ltd., Cat. No. P1110) for 15 min.

Fluorescence images were acquired using an Olympus IX83 fluorescence microscope equipped with a 20× objective. FITC-labeled conidia were detected using an excitation wavelength of 488 nm and an emission wavelength of 525 nm. For comparisons among strains, all images were acquired from randomly selected fields using identical exposure time, gain, and acquisition settings across all groups. Quantification was performed using ImageJ software, with at least 10 random fields analyzed per sample. Image were coded before analysis, and fluorescence quantification was performed by an investigator who was blinded to strain identity and experimental group allocation.

For invasion assays, A549 cells were seeded into 96-well plates and grown to confluence under the same culture conditions. Conidia were added at an MOI of 10 and incubated at 37°C with 5% CO_2_ for 6 h. Following incubation, cells were treated with nystatin (20 µg/mL) for 4 h to kill extracellular fungi, washed with PBS, and lysed with 0.25% Triton X-100 (Beijing APPLYGEN Gene Technology Co., Ltd., Cat. No. A1009) for 15 min. Cell lysates were serially diluted and plated onto Sabouraud dextrose agar (SDA) plates. After incubation at 37°C for 18–20 h, colonies were counted, and invasion rates were calculated based on the number of internalized conidia relative to the initial inoculum [41].

### Adhesion assay

Adhesion assays were performed to evaluate the ability of conidia to adhere to host cell surfaces. A549 cells were grown to confluence, after which conidia were added at a multiplicity of infection (MOI) of 10 and incubated at 37°C with 5% CO_2_ for 30 min. Unattached conidia were removed by washing with phosphate-buffered saline (PBS). Aspergillus minimal medium (AMM) was then added, and cells were incubated for 18–20 h to allow adhered conidia to germinate and form colony-forming units (CFUs). Colonies in each well were subsequently counted. To avoid overestimation due to colony fusion, only clearly separated and well-defined CFUs were included in the analysis. Adhesion efficiency was expressed as the ratio of recovered CFUs to the total number of conidia initially added per well. Each experiment included at least three independent biological replicates.

### Infection of the Galleria mellonella

Final-instar *Galleria mellonella* larvae were purchased from Beijing Anxinkang Technology Co., Ltd. (Beijing, China). Healthy larvae with uniform body size, light color, no visible melanization, and active movement were selected for infection experiments. Larvae weighing approximately 300 ± 30 mg were randomly assigned to experimental groups, with 16 larvae per group. Healthy final-instar *Galleria mellonella* larvae with uniform body size, light color, no visible melanization, and active movement were selected for infection experiments. Larvae weighing approximately 300 ± 30 mg were randomly assigned to experimental groups, with 16 larvae per group. A suspension containing 1 × 10^6^ conidia per larva was injected into the hemocoel through the last proleg using a microinjector. Control larvae were injected with an equal volume of 0.1% phosphate-buffered saline containing Tween 20 (PBST).

Following infection, larvae were incubated at 37°C in the dark, and survival and melanization were monitored at regular intervals. Larvae were considered dead when they failed to respond to tactile stimulation and exhibited extensive melanization. Each experiment was performed three independent times. Survival curves were generated using the Kaplan–Meier method, and statistical differences between groups were analyzed using the log-rank (Mantel–Cox) test [42].

### Propidium iodide staining

Propidium iodide (PI) staining was used to evaluate membrane permeability as previously described with minor modifications [43]. Fresh conidia were diluted in liquid minimal medium (MM) to 2 × 10^5 CFU/mL, and 500 μL of the suspension was added to each well of a 6-well plate. Cultures were incubated at 28°C for 24 h to allow mycelial formation. For azole-treated samples, mycelia were exposed to 0.25 μg/mL voriconazole (VOR) for 24 h before staining. Mycelia were then gently washed with PBS, incubated with PI (Beijing Solarbio Science & Technology Co., Ltd., Cat. No. IP5030) at a final concentration of 50 μg/mL for 15 min in the dark, and washed three times with PBS. Bright-field and fluorescence images were acquired using an Olympus IX83 fluorescence microscope under identical imaging settings. PI fluorescence intensity was quantified using ImageJ and normalized to the corresponding *CEA17Δku80* control. For each biological replicate, multiple randomly selected fields were analyzed, and the mean value of these fields was used for statistical analysis.

### Targeted quantification of ergosterol and hopane-3β,22-diol

Targeted quantification of ergosterol and Hopane-3β,22-diol was performed by gas chromatography–mass spectrometry (GC-MS) as previously described for fungal sterol analysis, with modifications [44]. Briefly, *A. fumigatus* mycelial samples were hydrolyzed at 70°C for 2 h and extracted twice with methyl tert-butyl ether (MTBE). 5α-cholestane was added as an internal standard. The combined organic phases were dried under nitrogen, reconstituted in methanol, and derivatized with pyridine and N,O-bis(trimethylsilyl)trifluoroacetamide containing 1% trimethylchlorosilane (BSTFA +1% TMCS) at 70°C for 60 min.

GC-MS analysis was performed using an Agilent 7890A/5975C GC-MS system equipped with an OPTIMA® 5 MS Accent capillary column (30 m × 0.25 mm × 0.25 μm). Helium was used as the carrier gas at a flow rate of 1 mL/min. The oven temperature program was as follows: 100°C for 1 min, increased to 250°C at 50°C/min and held for 3 min, then increased to 310°C at 4°C/min and held for 1 min. The injector, transfer line, and electron ionization source temperatures were set to 250°C, 260°C, and 230°C, respectively. Electron ionization was performed at 70 eV. Data were acquired in both full-scan and selected ion monitoring (SIM) modes over an m/z range of 50–600. Ergosterol and Hopane-3β,22-diol were quantified using calibration curves generated from authentic standards, and results were expressed as μg/g wet sample.

### Image quantification and statistical analysis

Fluorescence images were acquired using identical exposure and acquisition settings and analyzed using ImageJ software. Quantitative data are presented as mean ± SD unless otherwise indicated. Statistical analyses were performed using GraphPad Prism. For comparisons between two groups, two-tailed Student’s t-tests or Mann–Whitney U tests were used depending on data distribution and experimental design. For multiple-group comparisons, one-way ANOVA followed by Dunnett’s or Tukey’s multiple-comparison test was used as indicated in the figure legends. For colony growth assays, endpoint colony diameters at 96 h were compared between each deletion mutant and *CEA17Δku80* at the same temperature using one-way ANOVA followed by Dunnett’s multiple-comparison test. For RPMI-1640 liquid growth assays, endpoint OD600 values at 30 h were analyzed in the same manner. Survival curves were analyzed using the Kaplan–Meier method and compared by the log-rank (Mantel–Cox) test. A *p* value < 0.05 was considered statistically significant.

## Results

### Identification and comparative analysis of SHC-like triterpene cyclase genes in A. fumigatus

**Figure 1.**
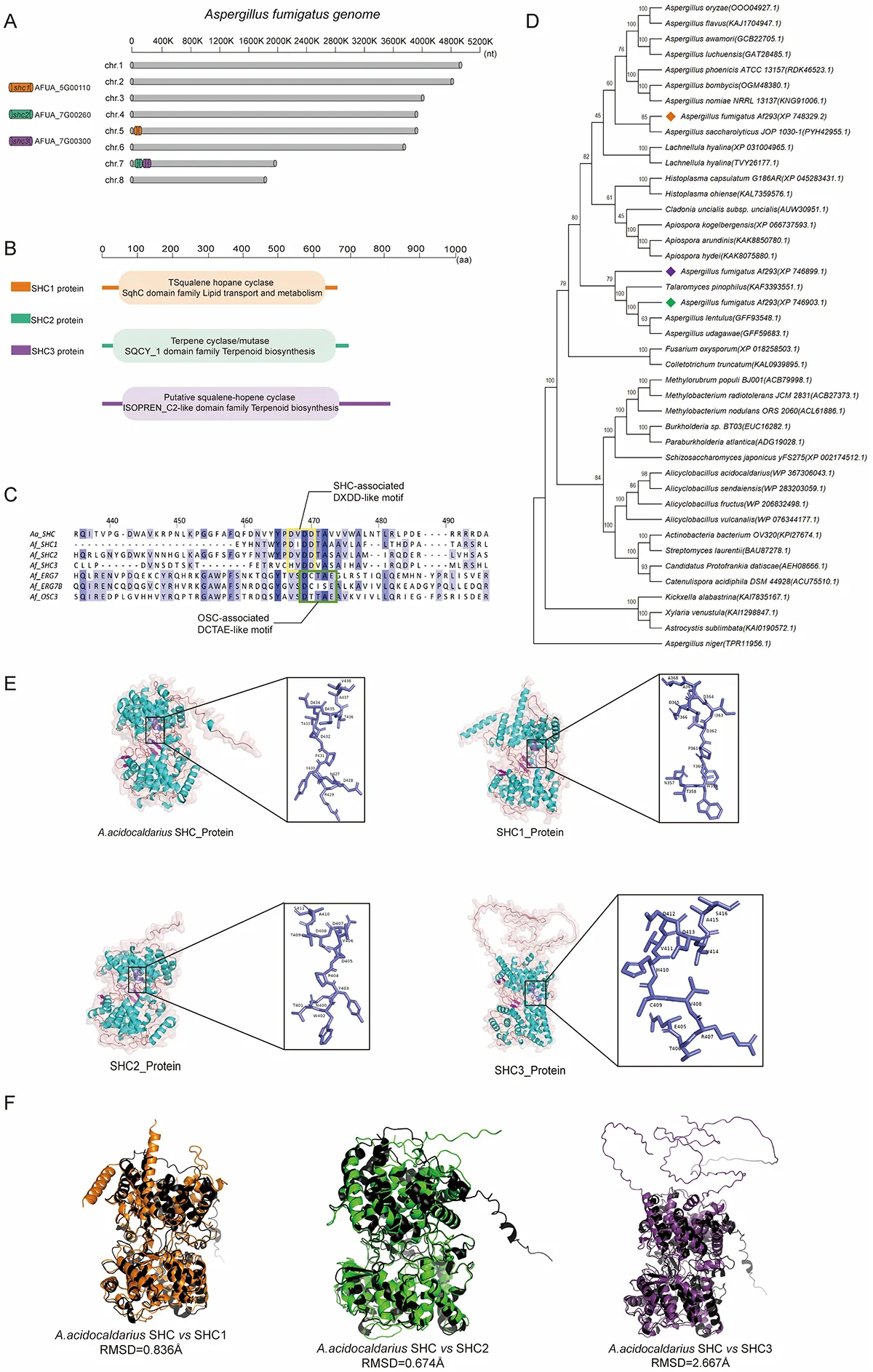
Identification and comparative analysis of SHC-like triterpene cyclase genes and proteins in *A. fumigatus*. (A) Genomic localization of sh*c1, shc2*, and *shc3* in the *A. fumigatus* Af293 genome. The BLASTp query coverage, percent identity, and E-values obtained using *A. acidocaldarius* SHC as the query are summarized in Table S3. (B) Conserved domain characterization of the three SHC-like triterpene cyclase proteins in *A. fumigatus*. (C) Local multiple sequence alignment of representative motif regions among *A. acidocaldarius* SHC, *A. fumigatus* SHC-like triterpene cyclase proteins, and selected OSC-like comparator proteins using ESPript 3.0. SHC-associated DXDD-like and OSC-associated DCTAE-like motif regions are highlighted. (D) Phylogenetic analysis of *A. fumigatus* SHC-like triterpene cyclase proteins, constructed using the maximum likelihood method in MEGA 11 with 1,000 bootstrap replicates. (E) Predicted three-dimensional structures of *A. fumigatus* SHC-like triterpene cyclase proteins generated by AlphaFold3 and visualized in PyMOL. (F) Structural superimposition of *A. fumigatus* SHC-like triterpene cyclase proteins with *A. acidocaldarius* SHC. The *A. acidocaldarius* SHC structure is shown in black, while *A. fumigatus* SHC-like triterpene cyclase proteins are shown in orange, green, and purple, respectively. RMSD values were calculated to estimate structural similarity.

SHC facilitates the cyclization of squalene into hopanoids in bacteria. Hopanoids operate in bacterial cell membranes analogous to sterols in fungal cell membranes, constituting a category of pentacyclic triterpenoids. The SHC protein from *A. acidocaldarius* is the earliest identified and most exemplary bacterial SHC [18]. To identify candidate SHC-like triterpene cyclase genes in *A. fumigatus*, we performed BLASTp analysis using the well-characterized squalene-hopene cyclase from *A. acidocaldarius* as the query against the predicted proteome of *A. fumigatus Af293*. This search identified AFUA_5G00110, AFUA_7G00260, and AFUA_7G00300 as the top three significant hits, providing a sequence-based rationale for focusing on these three candidates (Table S3). Based on these results and previous annotations, these genes were designated *shc1* (AFUA_5G00110), *shc2* (AFUA_7G00260), and *shc3* (AFUA_7G00300), respectively. Their chromosomal locations are shown in Figure 1(A). Notably, *shc2* and *shc3* are located close to each other on chromosome 7, suggesting that they may represent closely linked paralogous triterpene cyclase-like genes. Because fungal SHC-like triterpene cyclases may share sequence features with OSC/ERG7-like proteins, we also included AFUA_5G04080, AFUA_4G12040, and AFUA_4G14770 as bioinformatic comparators. These proteins were also identified as related triterpene cyclase hits in the BLASTp analysis but ranked below *shc1*, *shc2*, and *shc3* (Table S3). Therefore, they were included for comparative sequence analysis but were not selected for functional characterization in the present study.

We next analyzed the genomic contexts of the three candidate genes using antiSMASH. *shc1* was located in a predicted terpene region with high similarity to the reported fumihopaside-like biosynthetic gene cluster, whereas *shc2* and *shc3* were located together in a separate predicted terpene region on chromosome 7 that showed partial similarity to the fumihopaside-like cluster (Table S5). These results indicate that all three candidates are associated with terpene-related genomic regions, but *shc2* and *shc3* differ from *shc1* in their cluster context.

Protein domain analysis showed that the three candidates contain conserved triterpene cyclase-related domains (Figure 1(B)). Multiple sequence alignment of *A. acidocaldarius* SHC, the three *A. fumigatus* candidates, and representative OSC-like comparators revealed both conserved and divergent motif features, including differences in the DXDD-like and DCTAE-like regions (Figure 1(C)) [18,26]. Phylogenetic analysis showed that *shc2* and *shc3* clustered more closely with each other than with *shc1*, consistent with their adjacent genomic localization (Figure 1(D)). Structural prediction and superimposition with *A. acidocaldarius* SHC further showed that *shc1* and *shc2* retained broadly similar barrel-like folds, whereas *shc3* displayed greater divergence in the predicted catalytic region (Figure 1(E,F)). Together, these analyses suggest sequence, genomic-context, and structural divergence among the three SHC-like triterpene cyclase candidates in *A. fumigatus*.

### Deletion of individual shc genes causes limited effects on vegetative growth and development

Using the non-homologous end-joining-deficient *A. fumigatus* strain *CEA17Δku80* as the parental strain, we generated single-deletion mutants of *shc1*, *shc2*, and *shc3*. Correct replacement of the target loci was verified by diagnostic PCR, including target-gene PCR and 5'/3' junction PCR (Fig. S1A–D). RT-qPCR analysis further confirmed that the corresponding target-gene transcript was nearly undetectable in each deletion mutant (Fig. S1E–G). In addition, deletion of individual *shc* genes resulted in distinct changes in the expression of the remaining *shc* homologs, suggesting a transcriptional response within this gene family. The confirmed deletion mutants were then used for phenotypic analyses.

**Figure 2.**
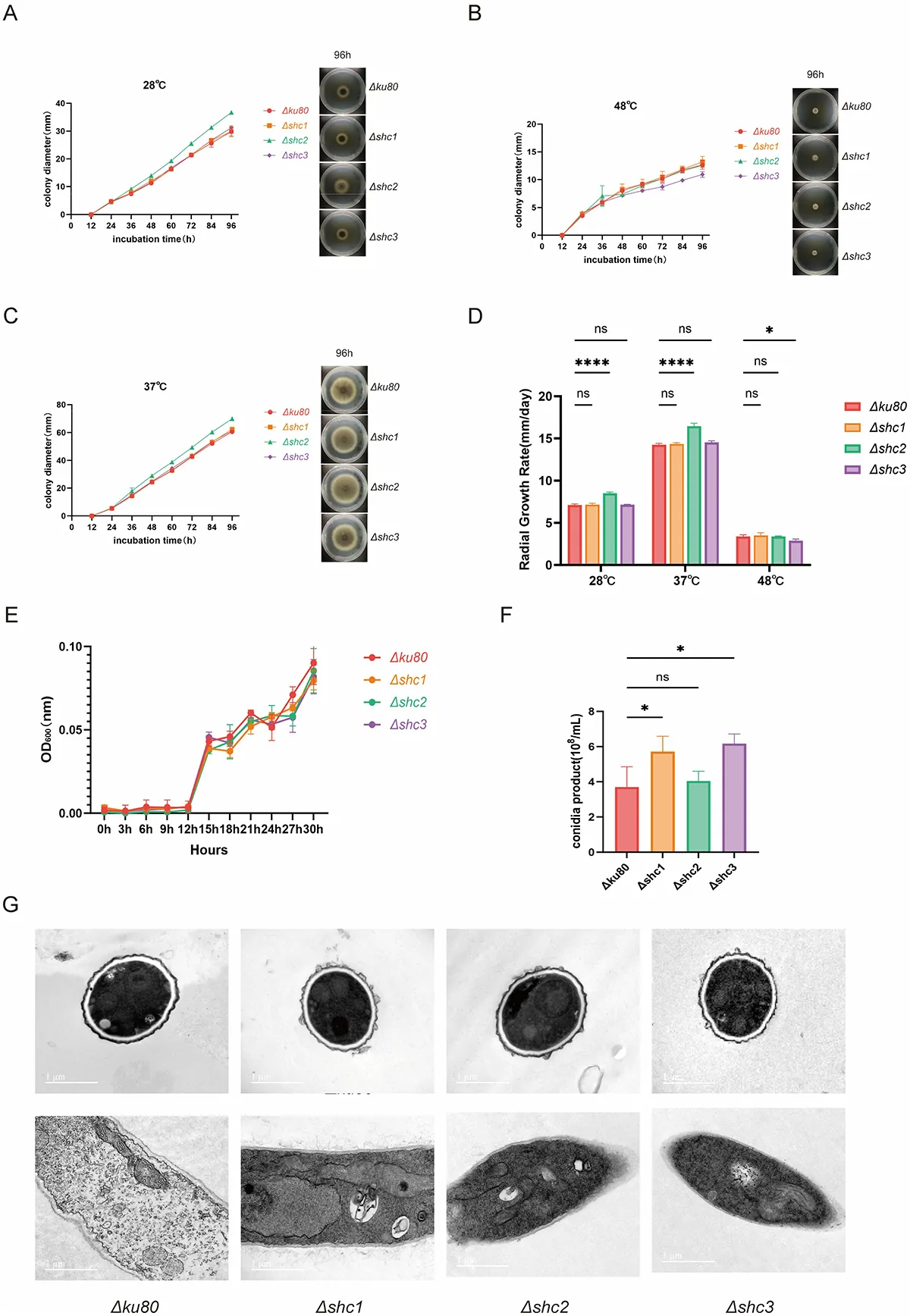
Effects of individual *shc* deletion on growth and development of *A. fumigatus*. (A–C) Colony growth curves and representative colony images of *CEA17Δku80*, Δ*shc1*, Δ*shc2*, and Δ*shc3* grown on AMM plates at 28°C, 37°C, and 48°C, respectively. Colony images were taken at 96 h. (D) Colony growth rates after 72 h on AMM medium at 28°C, 37°C, and 48°C. (E) Growth curves of *CEA17Δku80* and Δ*shc* mutants in RPMI-1640 liquid medium at 37°C, monitored by OD_600_. (F) Conidial production after 72 h of cultivation at 37°C on AMM media. (G) Morphological analysis of conidia and hyphae via transmission electron microscopy at equivalent magnifications for various strains. Data are shown as mean ± SD. ns, not significant; *, *p* < 0.05; ***, *p* < 0.001; ****, *p* < 0.0001.

We first measured colony growth on AMM plates at different temperatures. Overall, deletion of individual *shc* genes did not cause severe growth defects under the tested conditions (Figure 2(A–D)). However, Δ*shc2* showed a modest but statistically significant increase in colony diameter at 96 h compared with CEA17Δku80 at both 28°C and 37°C, whereas this difference was not observed at 48°C (Figure 2(A–D); Table S6). Δ*shc1* showed a slight increase in colony diameter at 37°C, while Δ*shc3* showed a modest reduction at 48°C (Table S6). These results indicate that deletion of individual *shc* genes has limited effects on vegetative growth, with Δ*shc2* displaying a temperature-dependent growth advantage under 28°C and 37°C conditions. We next assessed basal *shc* transcription in the *CEA17Δku80* parental strain under different temperature conditions. RT-qPCR analysis showed temperature-dependent changes in *shc* expression (Fig. S2A). However, these expression patterns in the parental strain did not directly match the growth phenotypes of the corresponding deletion mutants, indicating that basal transcript abundance alone is insufficient to explain the observed growth differences.

Growth in RPMI-1640 liquid medium was further monitored by OD_600_. In contrast to the modest differences observed on solid AMM medium, all strains showed broadly similar growth trends in RPMI-1640, and no significant differences in endpoint OD_600_ were detected between the deletion mutants and *CEA17Δku80* at 30 h (Figure 2(E); Table S7).

We next quantified asexual development and found that Δ*shc1* and Δ*shc3* produced more conidia than the parental strain under the tested condition, whereas Δ*shc2* did not show a comparable increase (Figure 2(F)). Furthermore, transmission electron microscopy revealed no obvious morphological abnormalities in conidia or hyphae of Δ*shc1*, Δ*shc2*, or Δ*shc3* compared with *CEA17Δku80* (Figure 2(G)). Taken together, these results suggest that individual *shc* deletions do not broadly impair growth or cellular morphology in *A. fumigatus*, but they can lead to limited and condition-dependent changes in colony expansion and conidiation.

### Deletion of shc1 mildly reduces azole susceptibility in A. fumigatus

**Figure 3.**
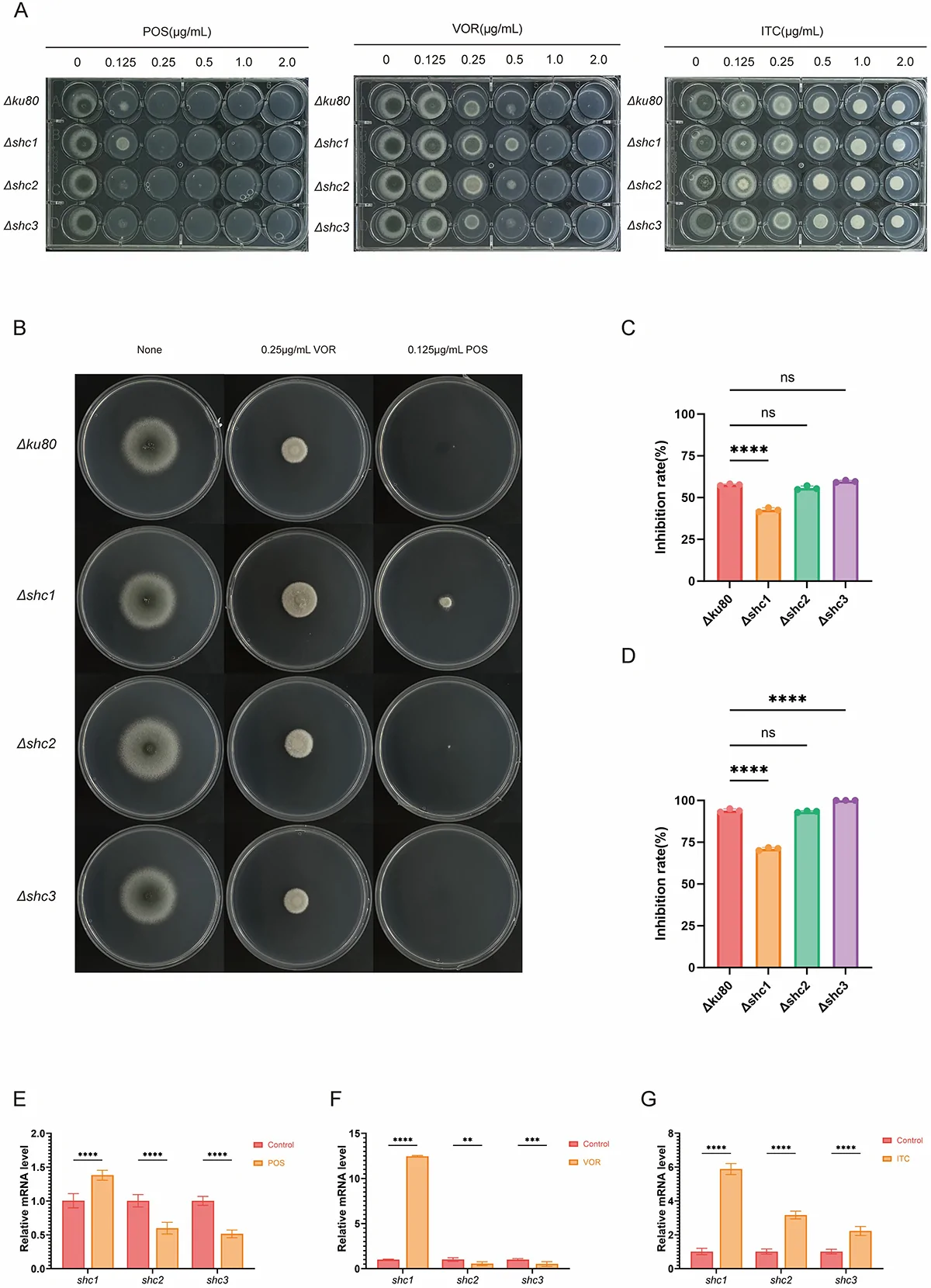
Effects of individual *shc* deletion on azole susceptibility in *A. fumigatus*. (A) Growth of CEA17Δku80, Δ*shc1*, Δ*shc2*, and Δ*shc3* on AMM plates supplemented with different concentrations of itraconazole (ITC), voriconazole (VOR), or posaconazole (POS). (B) Growth of the indicated strains after incubation at 37°C for 48 h in the presence of 0.125 µg/mL POS or 0.25 µg/mL VOR. (C, D) for subinhibitory azole assays, colony diameters were measured at the same incubation time point under azole-treated and drug-free conditions. For each strain, the drug-free condition of the same strain was used as its corresponding control. The inhibition rate was calculated as follows: inhibition rate (%) = [1 − (*D*_drug_/*D*_control_)] × 100, where *D*_drug_ represents the colony diameter under azole treatment and *D*_control_ represents the colony diameter of the corresponding drug-free control. (E-G) Relative expression levels of *shc1, shc2*, and *shc3* in the CEA17Δku80 parental strain after short-term exposure to ITC (E), VOR (F), or POS (G). Relative transcript levels were determined by RT-qPCR using *tub1* as the reference gene. For each gene, expression was normalized to the corresponding untreated control, which was set to 1. Data are shown as mean ± SD from at least three biological replicates. ns, not significant; *, *p* < 0.05; **, *p* < 0.01; ***, *p* < 0.001; ****, *p* < 0.0001.

The principal target of azole medication is the 14-α-demethylase encoded by *cyp51A* [45], a crucial enzyme in the conversion of lanosterol to ergosterol. Squalene, a precursor in the production of lanosterol, also serves as a substrate for SHC [26,46]. On AMM plates supplemented with azole antifungals, Δ*shc1* showed a modest growth advantage under VOR and POS treatment compared with CEA17Δku80 and the other deletion mutants (Figure 3(A)). Consistently, EUCAST-based susceptibility testing showed a twofold increase in the MICs of VOR and POS in Δ*shc1*, whereas Δ*shc2* and Δ*shc3* showed MIC values comparable to those of the parental strain (Table 1). We further evaluated fungal growth under subinhibitory concentrations of azoles. In the presence of 0.125 µg/mL POS or 0.25 µg/mL VOR, Δ*shc1* showed lower inhibition rates than CEA17Δku80, Δ*shc2*, and Δ*shc3* (Figure 3(B–D)), supporting a modest azole tolerance phenotype associated with loss of *shc1*.

We next assessed whether *shc* transcription was responsive to short-term azole exposure in the parental strain. RT-qPCR analysis showed that *shc1* expression was induced after exposure to ITC, VOR, and POS, whereas the responses of *shc2* and *shc3* were less pronounced and varied among azole treatments (Figure 3(E–G)).

To investigate whether SHC functions during the response of *A. fumigatus* to other stresses, we examined the growth of different *shc*-deficient strains under various cell wall disruptors, oxidative stress, and pH conditions. Under cell wall stress and pH stress conditions, no obvious phenotypic changes were observed in the deletion mutants compared with the parental strain, whereas Δ*shc2* showed increased tolerance to oxidative stress. These results suggest that individual *shc* deletions do not broadly impair cell wall integrity, oxidative stress responses, or pH adaptation under the tested conditions (Fig. S2B-F).

We next examined whether *shc* deletion was associated with changes in sterol/hopane-type triterpenoid metabolism and membrane permeability. Targeted GC-MS analysis showed that, in the Δshc1 batch, Hopane-3β,22-diol was markedly reduced in *Δshc1*, whereas ergosterol showed a modest increase compared with *CEA17Δku80* (Fig. S3A, B). Because targeted metabolite profiling was performed in two experimental batches, metabolite abundance was further normalized to the corresponding batch-matched *CEA17Δku80* control. In a separate batch, *Δshc2* and Δshc3 showed reduced relative abundance of both ergosterol and Hopane-3β,22-diol (Fig. S3C). These results indicate that individual *shc* deletions can alter sterol/hopane-type triterpenoid metabolic balance, with *Δshc1* showing a distinct pattern associated with its mild azole susceptibility phenotype.

PI staining was then used to assess membrane permeability. PI fluorescence intensity varied among *Δshc* mutants and *CEA17Δku80* after VOR treatment and under basal conditions (Fig. S3D–F). These data suggest that shc deletion is associated with changes in membrane-associated properties.

### Individual shc deletion induces distinct transcriptional remodeling patterns

**Figure 4.**
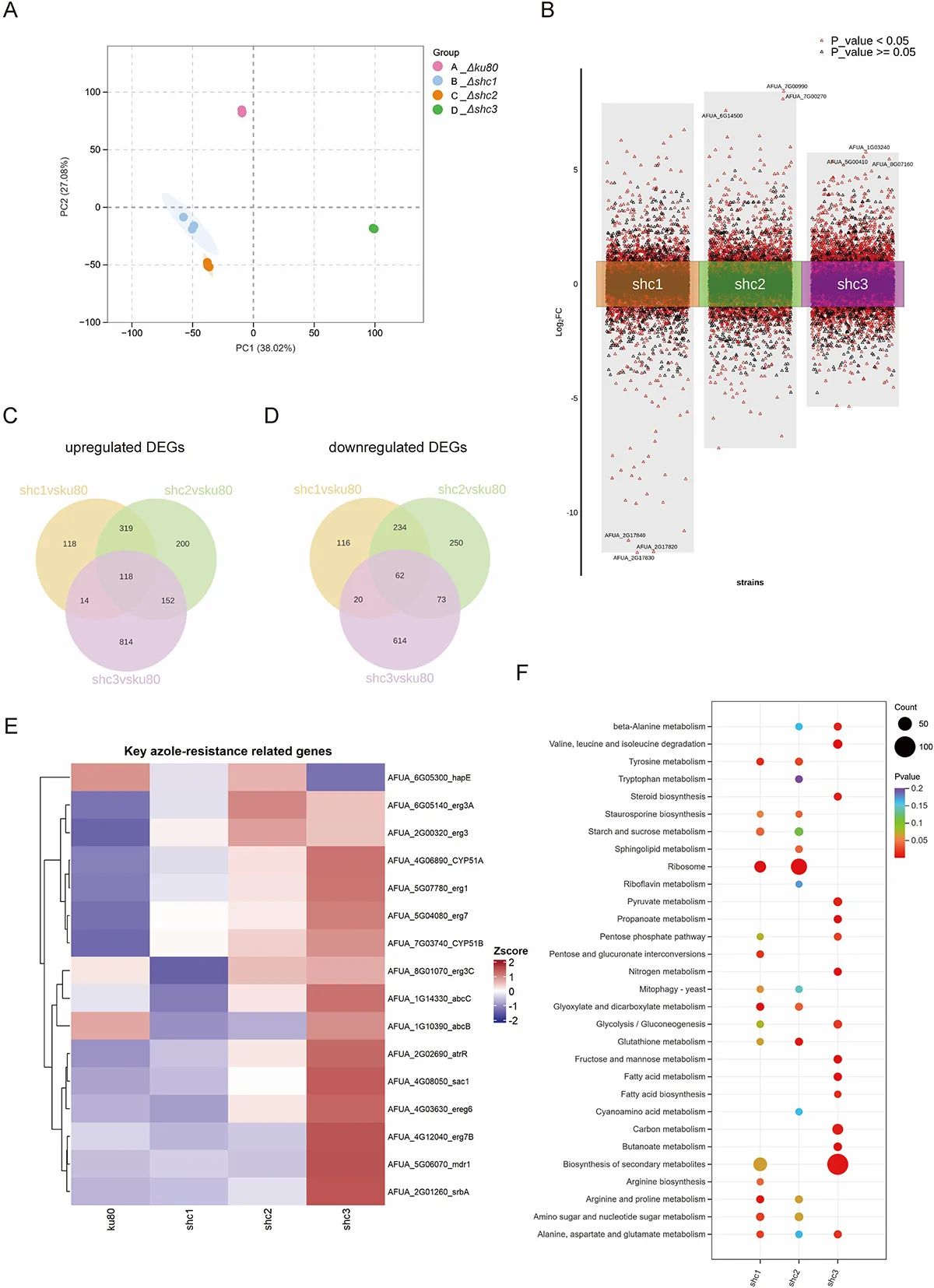
Transcriptomic assessment of *shc*-deficient mutants. (A) Principal component analysis of RNA-seq profiles from CEA17Δku80, Δ*shc1*, Δ*shc2*, and Δ*shc3*. (B) Volcano plots showing differentially expressed genes in each deletion mutant compared with CEA17Δku80. Red dots indicate significantly differentially expressed genes, and black dots indicate non-significant genes. (C, D) Venn diagram of differentially expressed genes (upregulated in C and downregulated in D) in three *shc* knockout lines relative to the parental line. (E) Heatmap showing the expression patterns of selected azole response-associated genes, including genes involved in ergosterol biosynthesis, transcriptional regulation, and multidrug efflux. Colors represent row-normalized Z-scores of gene expression levels, with red indicating relatively higher expression and blue indicating relatively lower expression. (F) KEGG pathway enrichment analysis of differentially expressed genes, where bubble size reflects the number of genes enriched in that pathway and color indicates the level of significance.

To analyze the molecular-level changes induced by *shc* deletion, we performed RNA-seq analysis on the parental strain and different *shc* deletion mutants. Principal component analysis showed good clustering of biological replicates and clear separation among strains, indicating that deletion of different *shc* genes produced distinct transcriptional profiles (Figure 4(A)). Differential expression analysis revealed that Δ*shc3* displayed the largest number of differentially expressed genes compared with *CEA17Δku80*, whereas Δ*shc1* and Δ*shc2* showed more limited transcriptional changes under the tested condition (Figure 4(B)). Venn diagram analysis further showed that Δ*shc3* contained the highest number of uniquely upregulated and downregulated genes, while Δ*shc1* and Δ*shc2* shared a subset of co-regulated genes (Figure 4(C,D)). These results suggest that loss of *shc3* induces broader transcriptional remodeling than loss of *shc1* or *shc2*.

KEGG enrichment analysis showed that DEGs in Δ*shc1* and Δ*shc2* were mainly enriched in pathways related to metabolism and protein synthesis, whereas DEGs in Δ*shc3* were more prominently enriched in pathways associated with secondary metabolism and metabolic reprogramming (Figure 4(F)). This pattern is consistent with the sequence, structural, and genomic-context divergence observed among the three SHC-like triterpene cyclase candidates. Because Δ*shc1* showed a mild reduction in azole susceptibility, we further selected representative genes in *A. fumigatus* closely associated with azole resistance or drug response for expression analysis. These genes primarily included *erg* series genes related to the ergosterol biosynthesis pathway, as well as key transcription factors *atrR*, *srbA*, and *hapE* involved in resistance regulation [45,47,48]. Additionally, we included genes associated with multidrug efflux transporters, such as *abcB*, *abcC*, and *mdr1* [49]. Several of these genes, including *cyp51A*, *atrR*, and *mdr1*, showed relatively higher expression in Δ*shc3* than in the other strains. However, this broader transcriptional activation in Δ*shc3* did not correspond to an increased MIC phenotype, suggesting that transcriptional induction of azole response-associated genes alone is insufficient to determine azole susceptibility. These findings indicate that *shc3* deletion triggers broad compensatory transcriptional remodeling, whereas the mild azole susceptibility shift observed in Δ*shc1* may involve more specific metabolic or membrane-associated changes (Figure 4(E)).

### Individual shc deletion alters early epithelial cell interactions and whole-host virulence differently

**Figure 5.**
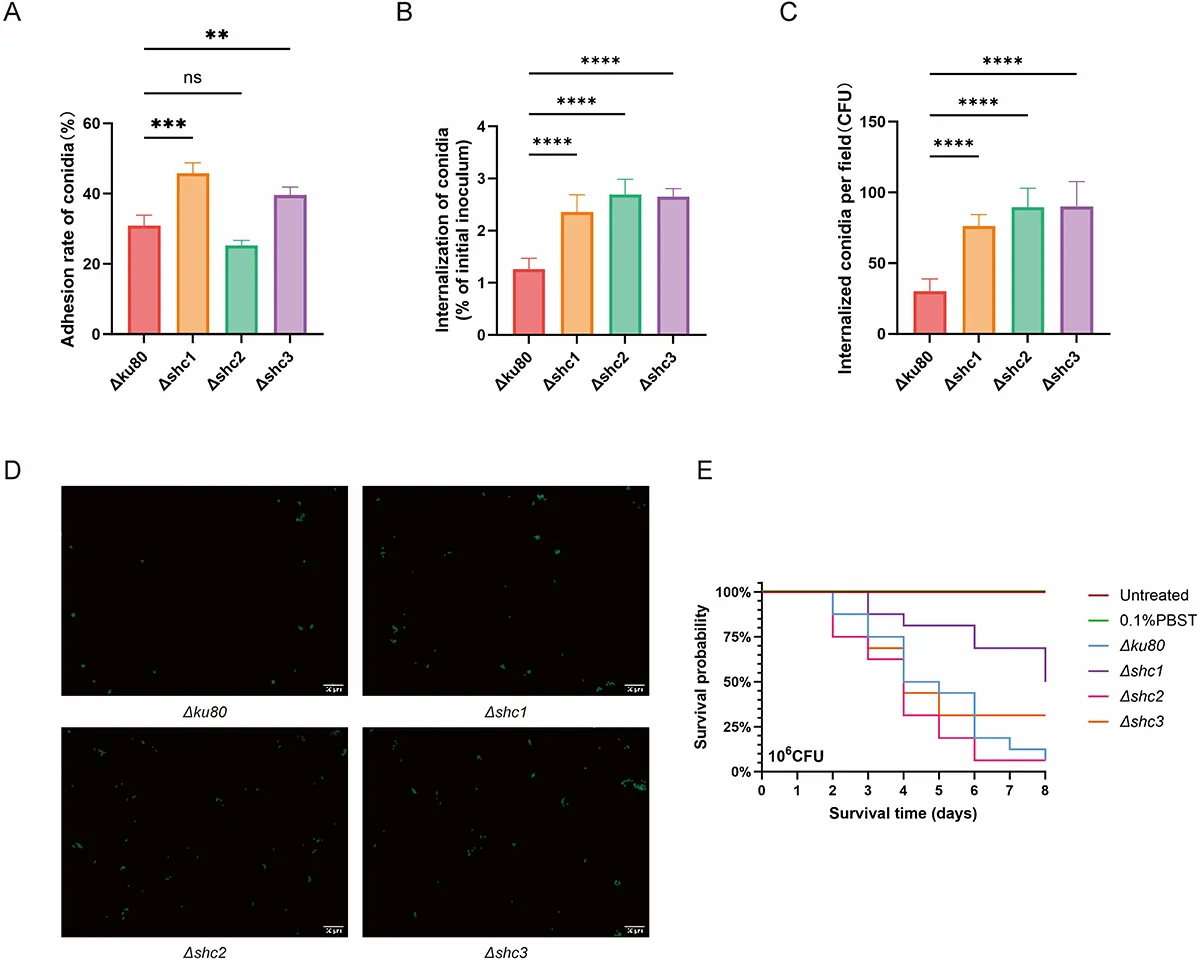
Effects of individual *shc* deletion on epithelial cell interaction and *Galleria mellonella* virulence. (A) Adhesion rates of several *shc* deletion strains and the parental strain to A549 cells. (B) Recovery of internalized conidia after infection of A549 cells, assessed using a nystatin-protection assay. (C) Quantity of FITC-labeled conidia per field of view following the infection of A549 cells. (D) Representative fluorescence microscopy pictures depicting FITC-labeled conidia infecting A549 cells. (E) Kaplan–Meier survival curves of *Galleria mellonella* larvae infected with conidia from CEA17Δku80 or Δ*shc* mutants. Data are shown as mean ± SD from at least three biological replicates. ns, *p* > 0.05; **, *p* < 0.01; ***, *p* < 0.001; ****, *p* < 0.0001.

To evaluate the impact of *shc* deletion on initial contacts between *A. fumigatus* and host cells, human alveolar epithelial cells (A549) were employed as a model to examine the adhesion and infection capabilities of conidia of various strains. Compared with *CEA17Δku80*, Δ*shc1* and Δ*shc3* showed increased adhesion to A549 cells, whereas Δ*shc2* did not show a comparable increase under the tested condition (Figure 5(A)). We next evaluated fungal internalization using a nystatin-protection assay. All three *shc* deletion mutants showed increased recovery of internalized conidia compared with *CEA17Δku80* (Figure 5(B)). Consistently, fluorescence microscopy of FITC-labeled conidia showed a higher intracellular conidial burden in A549 cells infected with the deletion mutants than in cells infected with the parental strain (Figure 5(C,D)). These results indicate that deletion of individual *shc* genes can enhance early epithelial cell-associated phenotypes, particularly adhesion and internalization.

The innate immunological response of the *Galleria mellonella* exhibits specific similarities to that of mammals [50]. To determine whether these in vitro host-cell interaction phenotypes were reflected in whole-host virulence, we further used the *Galleria mellonella* infection model. Kaplan–Meier survival analysis showed that larvae infected with Δ*shc1* survived significantly longer than those infected with *CEA17Δku80*, whereas Δ*shc2* and Δ*shc3* did not show significant differences from the parental strain (Figure 5(E)). Thus, the enhanced adhesion/internalization observed in the A549 model did not translate into increased virulence in the *Galleria* model. These findings suggest that individual *shc* deletions may differentially affect early epithelial interaction and overall host infection outcome.

### Elevated shc transcription is associated with azole-resistant clinical isolates

**Figure 6.**
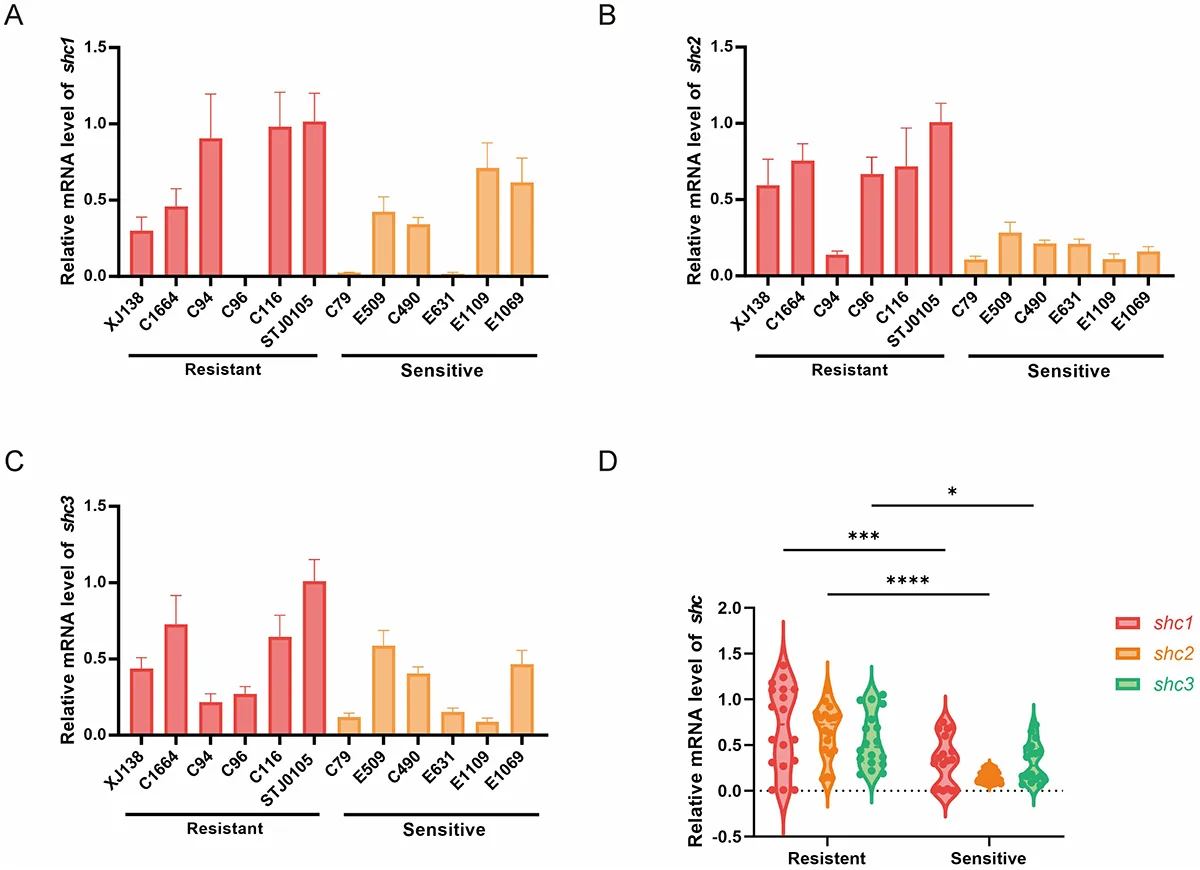
Expression profiles of clinical *A. fumigatus* isolates. (A-C) RT-qPCR analysis of relative transcript levels of *shc1* (A), *shc2* (B), and *shc3* (C) in 12 clinical *A. fumigatus* isolates, including six azole-resistant and six azole-susceptible isolates classified according to antifungal susceptibility profiles. MIC values and *cyp51A* mutations are listed in Table S8. Resistant isolates are shown in red, and susceptible isolates are shown in orange. (D) Expression data from the resistant and susceptible groups were aggregated into a violin plot, illustrating the distribution and variability of expression within each group. Bilateral Mann–Whitney U tests were employed for group comparisons. *, *p* < 0.05; ***, *p* < 0.001; ****, *p* < 0.0001.

To further evaluate the clinical relevance of the association between SHC and azole response, we included 12 clinical isolates of *A. fumigatus* and classified them into azole-resistant and sensitive groups based on antimicrobial susceptibility phenotypes (6 strains each; MICs are shown in Table S8). RT-qPCR analysis showed that the transcript levels of *shc1*, *shc2*, and *shc3* were higher in the azole-resistant group than in the susceptible group (Figure 6(A–C)). Pooled violin plot analysis further showed higher overall *shc* expression and greater inter-isolate variability in the resistant group (Figure 6(D)). These findings suggest an association between *shc* transcription and azole-resistant clinical backgrounds.

## Discussion

Azole antifungals exert their activity by interfering with ergosterol biosynthesis and remain important agents for the treatment and prevention of invasive aspergillosis. In this study, we systematically characterized three SHC-like triterpene cyclase genes in *A. fumigatus*, namely *shc1*, *shc2*, and *shc3*, focusing on their genetic features, phenotypic effects, transcriptional responses, and host interaction-related phenotypes. Our results showed that these three candidate genes differ in sequence features, genomic cluster contexts, and transcriptomic effects, suggesting that they are unlikely to represent simply redundant homologs but may instead exhibit a certain degree of functional differentiation. Among them, deletion of *shc1* caused a mild reduction in susceptibility to POS and VOR, reflected by an approximately twofold increase in MIC values and reduced inhibition rates, whereas deletion of *shc2* or *shc3* did not produce a similar azole susceptibility phenotype. Meanwhile, transcriptomic analysis showed that Δ*shc3* induced broader transcriptional remodeling, suggesting that different *shc* members may participate in metabolic adaptation and stress regulation in *A. fumigatus* through distinct layers of regulation.

An interesting observation is that Δ*shc1* displayed the most evident azole-related phenotype, although it did not show the most extensive transcriptomic changes. Conversely, Δ*shc3* did not exhibit a detectable MIC change but induced a larger number of differentially expressed genes, accompanied by relatively increased expression of azole response-associated genes such as *cyp51A*, *atrR*, and *mdr1*. This may be because MIC or drug-tolerance phenotypes are not determined solely by the transcriptional levels of individual response-associated genes, but may also be influenced by metabolic flux, membrane lipid composition, drug uptake and efflux, target-protein activity, and the overall physiological state of the cell. The broad transcriptional changes observed in Δ*shc3* may therefore reflect compensatory transcriptional remodeling or metabolic stress rather than a response that translates into a measurable MIC increase. Taken together, these observations suggest that the azole-related phenotype of *Δshc1* is unlikely to be explained simply by the extent of global transcriptional remodeling. Rather, *shc1* may affect azole response through a more specific influence on sterol/hopane-type triterpenoid balance and membrane-associated properties. This view is further supported by the targeted GC-MS and PI uptake analyses, although the direction of metabolic flux and the direct enzymatic products of each SHC-like protein remain to be determined.

Similarly, the basal transcript levels of *shc1*, *shc2*, and *shc3* in the *CEA17Δku80* parental strain changed in response to temperature, but these expression patterns did not directly predict the growth phenotypes of the corresponding deletion mutants. RT-qPCR analysis further showed that deletion of one *shc* gene altered the expression of the remaining homologs, suggesting a family-level transcriptional response. However, this response was not necessarily accompanied by restoration of sterol/hopane-type triterpenoid abundance, particularly in *Δshc2* and *Δshc3*. These findings indicate that the relationship among shc members is unlikely to represent a simple redundancy model, but may involve functional diversification and incomplete compensation at the metabolic level.

Research on SHC-like triterpene cyclase genes in *A. fumigatus* remains limited, but available evidence suggests that these enzymes may be connected to both secondary metabolism and membrane adaptation. Previous work has linked *shc1* to fumihopaside-like hopane-type triterpenoid biosynthesis, whereas the biological roles of *shc2* and *shc3* have not been systematically defined [24]. In fission yeast, SHC-derived hopanoid-like compounds can partially compensate for sterol limitation and help maintain membrane function, providing a broader biological context for a possible relationship between SHC-like enzymes, sterol availability, and membrane properties [22,23]. This context is relevant to azole response because azoles inhibit Cyp51-dependent ergosterol biosynthesis, thereby altering sterol composition, sterol intermediate accumulation, and membrane-associated functions [51,52].

**Figure 7.**
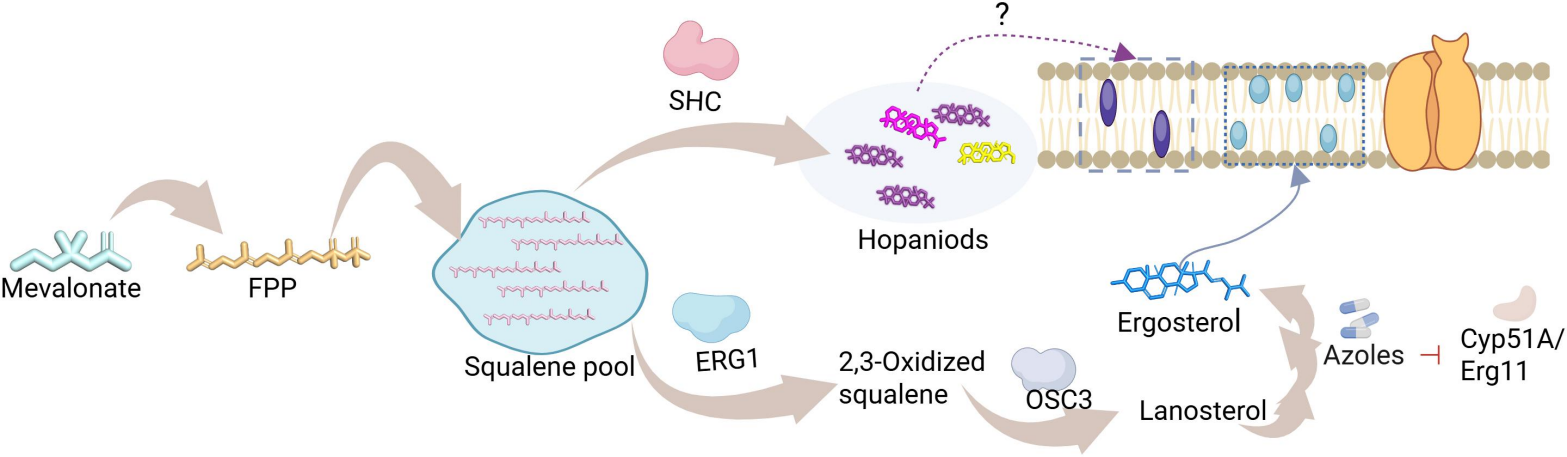
Working model of SHC-like triterpene cyclase-associated sterol/triterpenoid balance and membrane-associated azole response. Dashed arrows indicate proposed connections among squalene-associated metabolism, sterol/hopane-type triterpenoid balance, membrane-associated properties, and azole response, which require further experimental validation.

In the present study, the targeted GC-MS data support the idea that shc deletion affects this sterol/hopane-type triterpenoid balance rather than acting solely through transcriptional regulation. Among the three mutants, *Δshc1* showed the most azole-related phenotype and a distinct metabolite pattern, characterized by reduced Hopane-3β,22-diol and a modest increase in ergosterol relative to the parental strain. Together with the PI uptake results, these findings suggest that shc1 may influence azole response through membrane-associated metabolic adaptation. We therefore present this relationship as a working model (Figure 7). However, squalene levels, complete triterpenoid product profiles, direct metabolic flux, and membrane fluidity were not determined in this study, and the model requires further validation [21,53].

In the host-interaction assays, Δ*shc1* and Δ*shc3* showed increased adhesion to A549 cells, and all three deletion mutants exhibited enhanced internalization. However, in the *Galleria mellonella* infection model, only larvae infected with Δ*shc1* showed prolonged survival, indicating reduced overall lethality. This discrepancy suggests that the in vitro epithelial cell model and the in vivo infection model reflect different aspects of the pathogenic process. The A549 model mainly captures early host-cell interaction events, such as initial contact, adhesion, and internalization, whereas the *Galleria* model reflects the combined effects of in vivo survival, nutrient acquisition, stress tolerance, immune evasion, and tissue expansion [42,54]. Therefore, increased adhesion or internalization does not necessarily translate into increased overall virulence. If *shc1* deletion primarily alters conidial surface properties or the efficiency of early host-cell contact, it may enhance cell-level internalization while impairing sustained adaptation and expansion in the host, thereby resulting in increased A549 internalization but reduced lethality in the *Galleria* model.

The elevated transcription of *shc1*, *shc2*, and *shc3* in azole-resistant clinical isolates further suggests that these genes may be linked to the physiological state of resistant *A. fumigatus* backgrounds. Although most resistant isolates in this study carried known *cyp51A* resistance-associated mutations, the increased expression of *shc* genes may reflect additional adaptive features associated with azole-resistant strains, such as altered membrane homeostasis, metabolic remodeling, or stress-response activation. This interpretation is also consistent with our genetic data showing that *shc2* and *shc3* deletion did not alter MIC values despite their elevated expression in resistant isolates. Therefore, the clinical isolate data should be viewed as evidence of an association between *shc* transcription and azole-resistant backgrounds, rather than as proof that *shc* genes independently drive clinical resistance.

Although our findings provide new insight into the potential roles of SHC-like triterpene cyclase genes in *A. fumigatus*, several aspects remain to be further clarified. First, the functional analysis of *shc1*, *shc2*, and *shc3* was mainly based on single-gene deletion mutants. Complemented strains were not obtained, and double or triple *shc* deletion mutants were not successfully constructed; therefore, potential functional redundancy, synergistic effects, or compensatory relationships among the three members could not be fully evaluated. Second, although targeted GC-MS analysis of ergosterol and Hopane-3β,22-diol and PI uptake assays provided additional support for an association between *shc* deletion, sterol/hopane-type triterpenoid balance, and membrane-associated properties, the underlying biochemical mechanism remains incompletely defined. In particular, squalene levels, complete triterpenoid product profiles, direct metabolic flux, membrane lipid composition, and membrane fluidity were not determined in this study. Thus, the proposed mechanism by which shc1 affects membrane lipid homeostasis and azole susceptibility requires further validation through comprehensive lipidomics, flux analysis, structural identification of additional triterpenoid products, and membrane biophysical assays. The divergent results obtained from the A549 cell model and the Galleria mellonella infection model also warrant further investigation. These two models capture different aspects of host–pathogen interaction: the A549 model mainly reflects early epithelial adhesion and internalization, whereas the Galleria model incorporates additional in vivo factors, including fungal survival, stress tolerance, and host immune responses. Future studies incorporating time-course fungal burden analysis, host immune-response readouts, and infection models that better mimic the pulmonary microenvironment will be needed to clarify how *shc* deletion affects different stages of infection. Finally, although clinical isolate analysis showed elevated *shc* expression in resistant strains, the sample size was limited and most resistant isolates carried known *cyp51A* resistance-associated mutations. Thus, this result should currently be interpreted as an association between *shc* expression and azole-resistant clinical backgrounds rather than evidence that *shc* genes independently drive clinical azole resistance. Future studies with larger clinical isolate collections, different *cyp51A* backgrounds, non-*cyp51A*-mediated resistant isolates, and dynamic expression analysis under azole exposure will help further clarify the role of *shc* genes in clinical resistance adaptation.

## Supplementary Material

Supplementary_Material.docx

## Data Availability

The RNA-seq data generated in this study have been deposited in the NCBI Gene Expression Omnibus (GEO) under accession number GSE317610 and are publicly available at https://www.ncbi.nlm.nih.gov/geo/query/acc.cgi?acc=GSE317610. The numerical source data underlying the figures and tables are publicly available in Zenodo at https://doi.org/10.5281/zenodo.21157177 [55].

## References

[1] Alshareef F, Robson GD. Prevalence, persistence, and phenotypic variation of *aspergillus fumigatus* in the outdoor environment in manchester, UK, over a 2-year period. Med Mycol. 2014;52(4):367–19. doi: 10.1093/mmy/myu008 24719455

[2] VandenBergh MF, Verweij PE, Voss A. Epidemiology of nosocomial fungal infections: invasive aspergillosis and the environment. Diagn Microbiol Infect Dis. 1999;34(3):221–227. doi: 10.1016/s0732-8893(99)00026-7 10403102

[3] van de Veerdonk FL, Carvalho A, Wauters J, et al. *Aspergillus fumigatus* biology, immunopathogenicity and drug resistance. Nat Rev Microbiol. 2025;23(10):652–666. doi: 10.1038/s41579-025-01180-z 40316713

[4] Gu X, Hua Y-H, Zhang Y-D, et al. The pathogenesis of *aspergillus fumigatus*, host defense mechanisms, and the development of AFMP4 antigen as a vaccine. Pol J Microbiol. 2021;70(1):3–11. doi: 10.33073/pjm-2021-003 33815522 PMC8008755

[5] Earle K, Valero C, Conn DP, et al. Pathogenicity and virulence of *aspergillus fumigatus*. Virulence. 2023;14(1):2172264. doi: 10.1080/21505594.2023.2172264 36752587 PMC10732619

[6] Herrera S, Magyar U, Husain S. Invasive aspergillosis in the current era. Infect Dis Clin. 2025;39(1):e33–60. doi: 10.1016/j.idc.2025.01.002 40157842

[7] Denning DW. Global incidence and mortality of severe fungal disease. Lancet Infect Dis. 2024;24(7):e428–38. doi: 10.1016/S1473-3099(23)00692-8 38224705

[8] Lass-Flörl C, Steixner S. The changing epidemiology of fungal infections. Mol Aspects Med. 2023;94:101215. doi: 10.1016/j.mam.2023.101215 37804792

[9] Wichmann D, Hoenigl M, Koehler P, et al. Diagnosis and treatment of invasive pulmonary aspergillosis in critically ill intensive care patients: executive summary of the German national guideline (AWMF 113–005). Infection. 2025;53(4):1299–1310. doi: 10.1007/s15010-025-02572-2 40465080 PMC12316785

[10] Perlin DS, Rautemaa-Richardson R, Alastruey-Izquierdo A. The global problem of antifungal resistance: prevalence, mechanisms, and management. Lancet Infect Dis. 2017;17(12):e383–92. doi: 10.1016/S1473-3099(17)30316-X 28774698

[11] Wang C, Miller N, Vines D, et al. Azole resistance mechanisms and population structure of the human pathogen aspergillus fumigatus on retail plant products. Appl Environ Microbiol. 2024;90(5):e0205623. doi: 10.1128/aem.02056-23 38651929 PMC11107156

[12] Arendrup MC, Hare RK, Jørgensen KM, et al. Environmental hot spots and resistance-associated application practices for azole-resistant *aspergillus fumigatus* , Denmark, 2020–2023. Emerg Infect Dis. 2024;30(8):1531–1541. doi: 10.3201/eid3008.240096 38935978 PMC11286046

[13] van der Linden JWM, Arendrup MC, Warris A, et al. Prospective multicenter international surveillance of azole resistance in *aspergillus fumigatus*. Emerg Infect Dis. 2015;21(6):1041–1044. doi: 10.3201/eid2106.140717 25988348 PMC4451897

[14] Lockhart SR, Chowdhary A, Gold JAW. The rapid emergence of antifungal resistant human-pathogenic fungi. Nat Rev Microbiol. 2023;21(12):818–832. doi: 10.1038/s41579-023-00960-9 37648790 PMC10859884

[15] Burks C, Darby A, Londoño LG, et al. Azole-resistant aspergillus fumigatus in the environment: identifying key reservoirs and hotspots of antifungal resistance. PLoS Pathog. 2021;17(7):e1009711. doi: 10.1371/journal.ppat.1009711 34324607 PMC8321103

[16] Dladla M, Gyzenhout M, Marias G, et al. Azole resistance in aspergillus fumigatus- comprehensive review. Arch Microbiol. 2024;206(7):305. doi: 10.1007/s00203-024-04026-z 38878211

[17] Kimura M, Kushiro T, Shibuya M, et al. Protostadienol synthase from *aspergillus fumigatus*: functional conversion into lanosterol synthase. Biochem Biophys Res Commun. 2010;391(1):899–902. doi: 10.1016/j.bbrc.2009.11.160 19951700

[18] Siedenburg G, Jendrossek D. Squalene-hopene cyclases. Appl Environ Microbiol. 2011;77(12):3905–3915. doi: 10.1128/AEM.00300-11 21531832 PMC3131620

[19] Ochs D, Kaletta C, Entian KD, et al. Cloning, expression, and sequencing of squalene-hopene cyclase, a key enzyme in triterpenoid metabolism. J Bacteriol. 1992;174(1):298–302. doi: 10.1128/jb.174.1.298-302.1992 1729216 PMC205708

[20] Mangiarotti A, Genovese DM, Naumann CA, et al. Hopanoids, like sterols, modulate dynamics, compaction, phase segregation and permeability of membranes. Biochim Biophys Acta Biomembr. 2019;1861(12):183060. doi: 10.1016/j.bbamem.2019.183060 31499020

[21] Sáenz JP, Grosser D, Bradley AS, et al. Hopanoids as functional analogues of cholesterol in bacterial membranes. Proc Natl Acad Sci U S A. 2015;112(38):11971–11976. doi: 10.1073/pnas.1515607112 26351677 PMC4586864

[22] Rao BD, Gomez-Gil E, Peter M, et al. Horizontal acquisition of prokaryotic hopanoid biosynthesis reorganizes membrane physiology driving lifestyle innovation in a eukaryote. Nat Commun. 2025;16(1):3291. doi: 10.1038/s41467-025-58515-w 40195311 PMC11976957

[23] Bouwknegt J, Wiersma SJ, Ortiz-Merino RA, et al. A squalene–hopene cyclase in *schizosaccharomyces japonicus* represents a eukaryotic adaptation to sterol-limited anaerobic environments. Proc Natl Acad Sci U S A. 2021;118(32):e2105225118. doi: 10.1073/pnas.2105225118 34353908 PMC8364164

[24] Ma K, Zhang P, Tao Q, et al. Characterization and biosynthesis of a rare fungal hopane-type triterpenoid glycoside involved in the antistress property of *aspergillus fumigatus*. Org Lett. 2019;21(9):3252–3256. doi: 10.1021/acs.orglett.9b00984 30977375

[25] Kaneko I, Terasawa Y, Hoshino T. Squalene-hopene cyclase: mechanistic insights into the polycyclization cascades of squalene analogs bearing ethyl and hydroxymethyl groups at the C-2 and C-23 positions. Chem Weinh Bergstr Ger. 2018;24(43):11139–11157. doi: 10.1002/chem.201801668 29732636

[26] Hoshino T, Sato T. Squalene-hopene cyclase: catalytic mechanism and substrate recognition. Chem Commun Camb Engl. 2002;2002(4):291–301. doi: 10.1039/b108995c 12120044

[27] Hammer SC, Syrén P-O, Seitz M, et al. Squalene hopene cyclases: highly promiscuous and evolvable catalysts for stereoselective CC and CX bond formation. Curr Opin Chem Biol. 2013;17(2):293–300. doi: 10.1016/j.cbpa.2013.01.016 23485581

[28] Chen H, Zhang Y, Lee MY, et al. The key structure and self-stabilization mechanism of water-soluble interfacial squalene-hopene cyclase. Int J Biol Macromol. 2025;305(2):141340. doi: 10.1016/j.ijbiomac.2025.141340 39986529

[29] Liu Z, Zhang Y, Sun J, et al. A novel soluble squalene-hopene cyclase and its application in efficient synthesis of hopene. Front Bioeng Biotechnol. 2020;8:426. doi: 10.3389/fbioe.2020.00426 32478051 PMC7232578

[30] Hu M, Li Z, Li D, et al. Long terminal repeat retrotransposon Afut4 promotes azole resistance of *aspergillus fumigatus* by enhancing the expression of *sac1* gene. Antimicrob Agents Chemother. 2021;65(12):e0029121. doi: 10.1128/AAC.00291-21 34516252 PMC8597758

[31] da Silva Ferreira ME, Mrvz K, Savoldi M, et al. The akuBKU80 mutant deficient for nonhomologous end joining is a powerful tool for analyzing pathogenicity in *aspergillus fumigatus*. Eukaryot Cell. 2006;5(1):207–211. doi: 10.1128/EC.5.1.207-211.2006 16400184 PMC1360264

[32] Szewczyk E, Nayak T, Oakley CE, et al. Fusion PCR and gene targeting in *aspergillus nidulans*. Nat Protoc. 2006;1(6):3111–3120. doi: 10.1038/nprot.2006.405 17406574

[33] Gravelat FN, Askew DS, Sheppard DC. Targeted gene deletion in *aspergillus fumigatus* using the hygromycin-resistance split-marker approach. Methods Mol Biol Clifton NJ. 2012;845:119–130. doi: 10.1007/978-1-61779-539-8_8 22328371

[34] Furukawa T, van Rhijn N, Fraczek M, et al. The negative cofactor 2 complex is a key regulator of drug resistance in *aspergillus fumigatus*. Nat Commun. 2020;11(1):427. doi: 10.1038/s41467-019-14191-1 31969561 PMC7194077

[35] Zhang H, Ni H, Tao X, et al. Oxidoreductase gene *fabG* contributes to fungal development, cell wall integrity, and virulence in *aspergillus fumigatus*. Microbiol Spectr. 2026;14(1):e0209225. doi: 10.1128/spectrum.02092-25 41230995 PMC12772249

[36] Arendrup MC, Guinea J, Cuenca-Estrella M, et al. Eucast definitive document e.Def 9.3. 2015.

[37] Guinea J. Updated EUCAST clinical breakpoints against aspergillus, implications for the clinical microbiology laboratory. J Fungi Basel Switz. 2020;6(4):343. doi: 10.3390/jof6040343 PMC7762142 33291313

[38] Bustin SA, Benes V, Garson JA, et al. The MIQE guidelines: minimum information for publication of quantitative real-time PCR experiments. Clin Chem. 2009;55(4):611–622. doi: 10.1373/clinchem.2008.112797 19246619

[39] Livak KJ, Schmittgen TD. Analysis of relative gene expression data using real-time quantitative PCR and the 2(-delta delta C(T)) method. Methods San Diego Calif. 2001;25(4):402–408. doi: 10.1006/meth.2001.1262 11846609

[40] Persat F, Noirey N, Diana J, et al. Binding of live conidia of *aspergillus fumigatus* activates in vitro-generated human langerhans cells via a lectin of galactomannan specificity. Clin Exp Immunol. 2003;133(3):370–377. doi: 10.1046/j.1365-2249.2003.02222.x 12930363 PMC1808778

[41] Zhang X, He D, Gao S, et al. iTRAQ‑based proteomic analysis of the interaction of A549 human lung epithelial cells with Aspergillus fumigatus conidia. Mol Med Rep. 2020;22(6):4601–4610. doi: 10.3892/mmr.2020.11582 33174000 PMC7646843

[42] Maurer E, Browne N, Surlis C, et al. *Galleria mellonella* as a host model to study *aspergillus terreus* virulence and amphotericin B resistance. Virulence. 2015;6(6):591–598. doi: 10.1080/21505594.2015.1045183 26107350 PMC4720272

[43] Meng F, Liu X, Li C, et al. Hinokitiol inhibits *aspergillus fumigatus* by interfering with the cell membrane and cell wall. Front Microbiol. 2023;14:1132042. doi: 10.3389/fmicb.2023.1132042 37113218 PMC10128913

[44] Alcazar-Fuoli L, Mellado E, Garcia-Effron G, et al. Ergosterol biosynthesis pathway in *aspergillus fumigatus*. Steroids. 2008;73(3):339–347. doi: 10.1016/j.steroids.2007.11.005 18191972

[45] Diaz-Guerra TM, Mellado E, Cuenca-Estrella M, et al. A point mutation in the 14alpha-sterol demethylase gene *cyp51A* contributes to itraconazole resistance in *aspergillus fumigatus*. Antimicrob Agents Chemother. 2003;47(3):1120–1124. doi: 10.1128/AAC.47.3.1120-1124.2003 12604551 PMC149335

[46] Song L, Wang S, Zou H, et al. Regulation of ergosterol biosynthesis in pathogenic fungi: opportunities for therapeutic development. Microorganisms. 2025;13(4):862. doi: 10.3390/microorganisms13040862 40284698 PMC12029249

[47] Hagiwara D, Miura D, Shimizu K, et al. A novel Zn2-Cys6 transcription factor *AtrR* plays a key role in an azole resistance mechanism of *aspergillus fumigatus* by co-regulating *cyp51A* and *cdr1B* expressions. PLoS Pathog. 2017;13(1):e1006096. doi: 10.1371/journal.ppat.1006096 28052140 PMC5215518

[48] Camps SMT, Dutilh BE, Arendrup MC, et al. Discovery of a *HapE* mutation that causes azole resistance in *aspergillus fumigatus* through whole genome sequencing and sexual crossing. PLOS ONE. 2012;7(11):e50034. doi: 10.1371/journal.pone.0050034 23226235 PMC3511431

[49] Fraczek MG, Bromley M, Buied A, et al. The *cdr1B* efflux transporter is associated with non-cyp51a-mediated itraconazole resistance in *aspergillus fumigatus*. J Antimicrob Chemother. 2013;68(7):1486–1496. doi: 10.1093/jac/dkt075 23580559

[50] Tsai C-Y, Loh JMS, Proft T. *Galleria mellonella* infection models for the study of bacterial diseases and for antimicrobial drug testing. Virulence. 2016;7(3):214–229. doi: 10.1080/21505594.2015.1135289 26730990 PMC4871635

[51] Rosam K, Monk BC, Lackner M. Sterol 14α-demethylase ligand-binding pocket-mediated acquired and intrinsic azole resistance in fungal pathogens. J Fungi. 2020;7(1):1. doi: 10.3390/jof7010001 PMC7822023 33374996

[52] Alcazar-Fuoli L, Mellado E. Ergosterol biosynthesis in *aspergillus fumigatus*: its relevance as an antifungal target and role in antifungal drug resistance. Front Microbiol. 2013;3. doi: 10.3389/fmicb.2012.00439 PMC3541703 23335918

[53] Belin BJ, Busset N, Giraud E, et al. Hopanoid lipids: from membranes to plant–bacteria interactions. Nat Rev Microbiol. 2018;16(5):304–315. doi: 10.1038/nrmicro.2017.173 29456243 PMC6087623

[54] Wasylnka JA, Moore MM. *Aspergillus fumigatus* conidia survive and germinate in acidic organelles of A549 epithelial cells. J Cell Sci. 2003;116(8):1579–1587. doi: 10.1242/jcs.00329 12640041

[55] Ye K. Functional characterization of SHC-like triterpene cyclase genes in azole response and virulence-related traits of *aspergillus fumigatus*. 2026. doi: 10.5281/zenodo.21157177 PMC13556965 42700385

