# Supplementary material for: Functional characterization of SHC-like triterpene cyclase genes in azole response and virulence-related traits of Aspergillus fumigatus
Source: Virulence. 2026 Sep 5;17(1):2715234. doi: 10.1080/21505594.2026.2715234 (PMC13556965; doi:10.1080/21505594.2026.2715234)
Supplement: Supplementary file 1 [file KVIR_A_2715234_SM4585.docx]

Table S1 **Key reagents and materials used in this study**

| **Reagent** | **Supplier** | **Catalog No.** |
| --- | --- | --- |
| Fungal genomic DNA extraction kit | Hangzhou Bioer Technology Co.,Ltd. | BSC14M1 |
| TransZol Up Plus RNA Kit | TransGen Biotech Co., Ltd | ER501-01-V2 |
| TransScript All-in-one First-Strand cDNA Synthesis SuperMix for qPCR | TransGen Biotech Co., Ltd | AT341 |
| TransStart Top Green qPCR SuperMix | TransGen Biotech Co., Ltd | AQ131 |
| 2 × TransStart FastPfu Fly PCR SuperMix | TransGen Biotech Co., Ltd | AS231 |
| RPMI-1640 medium | Thermo Fisher Scientific, Inc | 31800022 |
| F-12K medium | Beijing Solarbio Science & Technology Co.,Ltd. | LA1320 |
| 3% BSA | Beijing Biotopped Technology Co., Ltd | Top1002 |
| MOPS | Beijing Biotopped Technology Co., Ltd | M6420T |
| Voriconazole | TCI (Shanghai) Development Co., Ltd. | V0116 |
| Itraconazole | Beijing Solarbio Science & Technology Co.,Ltd. | I8250 |
| Posaconazole | Anhui Senrise Technologies Co., Ltd. | E1200070050 |
| Hygromycin B | Beijing Coolaber Technology Co.,Ltd. | CH6361 |
| Nystatin | Beijing Solarbio Science & Technology Co., Ltd. | N8040 |
| Paraformaldehyde | Beijing Solarbio Science & Technology Co., Ltd. | P1110 |
| Triton X-100 | Beijing APPLYGEN Gene Technology Co., Ltd. | A1009 |
| FITC | Beijing Solarbio Science & Technology Co., Ltd. | CA1620 |
| PBS | Wuhan Servicebio Technology Co., Ltd | G4207 |

Table S2 **Primers used for construction and validation of *shc* knockout lines**

| No. | Primer | Sequence (5'→3') |
| --- | --- | --- |
| 1 | *Δshc1-up-F* | CTCACAAGCCGCATAAAAGTCC |
| 2 | *Δshc1-up-R* | GAGCTCCAGCTTTTGTTCCTCAACCGTCCGTCTCGTTCC |
| 3 | *Δshc1-hph-F* | GGAACGAGACGGACGGTTGAGGAACAAAAGCTGGAGCTC |
| 4 | *Δshc1-hph-R* | TGAACCAAACAAGACCAGCAGATGTCGACGGTATCGATAAGCTT |
| 5 | *Δshc1-dw-F* | AAGCTTATCGATACCGTCGACATCTGCTGGTCTTGTTTGGTTCA |
| 6 | *Δshc1-dw-R* | CGAATGCGAAGGCTAATGTGA |
| 7 | *Δshc1-upcheck-F* | CCGTCATTTCCTTCCTCACAAC |
| 8 | *Δshc1-upcheck-R* | TCACATTCTCCTTCGCTTACTGC |
| 9 | *Δshc1-dwcheck-F* | TCTCCTATGAGTCGTTTACCCAGAA |
| 10 | *Δshc1-dwcheck-R* | CGGCTGAGACAATAAAAGCACAATA |
| 11 | *Δshc2-up-F* | AAGACGTGCTTTGGCTTCACC |
| 12 | *Δshc2-up-R* | CAGAGCTCCAGCTTTTGTTCCCGCGAGGGTCAATTGGCAT |
| 13 | *Δshc2-hph-F* | ATGCCAATTGACCCTCGCGGGAACAAAAGCTGGAGCTCTG |
| 14 | *Δshc2-hph-R* | CTCCGTACGTTATAGCTAGATCCATGTCGACGGTATCGATAAGCTTG |
| 15 | *Δshc2-dw-F* | CAAGCTTATCGATACCGTCGACATGGATCTAGCTATAACGTACGGAG |
| 16 | *Δshc2-dw-R* | TTGATCGCATTTGGTTGATTG |
| 17 | *Δshc2-upcheck-F* | CTGTCCTGGGATCACCCGTAT |
| 18 | *Δshc2-upcheck-R* | CGGTGTCGTCCATCACAGTTT |
| 19 | *Δshc2-dwcheck-F* | GATCGTTATGTTTATCGGCACTTT |
| 20 | *Δshc2-dwcheck-R* | CCTGGCGGAAGTTGATTTAGAAT |
| 21 | *Δshc3-up-F* | AAGGTCATGCCCTGCGTTAT |
| 22 | *Δshc3-up-R* | CAGAGCTCCAGCTTTTGTTCCATGTATATCTACACCGTCGTGCAA |
| 23 | *Δshc3-hph-F* | TTGCACGACGGTGTAGATATACATGGAACAAAAGCTGGAGCTCTG |
| 24 | *Δshc3-hph-R* | CTTTGAGTTCGTTTTCTCTGTGCTGTCGACGGTATCGATAAGCTTG |
| 25 | *Δshc3-dw-F* | CAAGCTTATCGATACCGTCGACAGCACAGAGAAAACGAACTCAAAG |
| 26 | *Δshc3-dw-R* | CACGGGCAGAGGACTACACA |
| 27 | *Δshc3-upcheck-F* | GCCTTTGGTATCCAGTATGC |
| 28 | *Δshc3-upcheck-R* | CAGAAGAAGATGTTGGCGAC |
| 29 | *Δshc3-dwcheck-F* | CCTGGGTTCGCAAAGATAAT |
| 30 | *Δshc3-dwcheck-R* | GACTGATTGGCTAGAACGGA |
| 31 | *qpcr-shc1-F* | CTCCAAATCATCAACACCG |
| 32 | *qpcr-shc1-R* | AGTCCGCACAGAACAAGG |
| 33 | *qpcr-shc2-F* | TTCAACGGGGAATGGAGTAT |
| 34 | *qpcr-shc2-R* | AAAGTAATGGGCGTAGAGTG |
| 35 | *qpcr-shc3-F* | ATCGGAGTGTTGCGTTCA |
| 36 | *qpcr-shc3-R* | CCAGAGTCGTGCGTAGAG |
| 37 | *qpcr-tub1-F* | CTGTATCGACAACGAGGCTCT |
| 38 | *qpcr-tub1-R* | AGTTGAGCTGACCAGGGAAA |

Table S3 **BLASTp hits obtained using *Alicyclobacillus acidocaldarius* SHC as the query against the predicted proteome of *Aspergillus fumigatus Af293*.**

| Study name | Locus  Tag | Accession | Protein Annotation | Query coverage (%) | Percent identity (%) | E-value |
| --- | --- | --- | --- | --- | --- | --- |
| *shc1* | AFUA_5G00110 | XP_748329.2 | squalene hopane cyclase afumA | 98 | 36.04 | 5e-123 |
| *shc2* | AFUA_7G00260 | XP_746903.1 | terpene cyclase/mutase family protein | 94 | 39.78 | 1e-140 |
| *shc3* | AFUA_7G00300 | XP_746899.1 | putative squalene-hopene-cyclase | 97 | 25.84 | 1e-47 |
| *erg7* | AFUA_5G04080 | XP_077660888.1 | terpene cyclase/mutase family protein | 90 | 27.12 | 4e-41 |
| *erg7B* | AFUA_4G12040 | XP_751627.1 | terpene cyclase/mutase family protein | 89 | 24.05 | 2e-33 |
| *osc3* | AFUA_4G14770 | XP_751356.1 | oxidosqualene cyclase helA | 96 | 26.66 | 3e-45 |

Table S4 **Af293 AFUA identifiers, related A1163/AFUB orthologs, and deletion targets analyzed in the *CEA17Δku80* background**

| Study name | Af293 AFUA Locus Tag | Protein Accession | Related A1163/AFUB Ortholog | Usage in this study |
| --- | --- | --- | --- | --- |
| *shc1* | AFUA_5G00110 | XP_748329.2 | AFUB_071550 | deletion target in *CEA17Δku80* |
| *shc2* | AFUA_7G00260 | XP_746903.1 | AFUB_086820 |  |
| *shc3* | AFUA_7G00300 | XP_746899.1 | AFUB_086860 |  |

Table S5 **Predicted secondary metabolite gene-cluster contexts of *shc1, shc2, and shc3* based on antiSMASH analysis.**

| **Gene** | **Af293 locus** | **A1163 ortholog** | **antiSMASH region** | **Predicted Cluster type** | **Most similar known cluster** | **Similarity score/confidence** | **Interpretation** |
| --- | --- | --- | --- | --- | --- | --- | --- |
| *shc1* | AFUA_5G00110 | AFUB_071550 | Region 5.1 | terpene | fumihopaside A / compound 3 / 21-β-H-hopane-3β,22-diol | High | Located in a predicted terpene region highly similar to the fumihopaside/hopane-type triterpenoid BGC |
| *shc2* | AFUA_7G00260 | AFUB_086820 | Region 7.2 | terpene | fumihopaside A / compound 3 / 21-β-H-hopane-3β,22-diol | similarity score 0.54 | Located in a predicted terpene region with partial similarity to the fumihopaside-like BGC |
| *shc3* | AFUA_7G00300 | AFUB_086870 | Region 7.2 | terpene | fumihopaside A / compound 3 / 21-β-H-hopane-3β,22-diol | similarity score 0.54 | Located in the same predicted terpene region as *shc2*, with partial similarity to the fumihopaside-like BGC |

Table S6 **Statistical comparison of colony diameters at 96 h among *CEA17Δku80* and *Δshc* mutants at different temperatures.**

| **Temperature** | **Strain** | **Colony diameter (mm), mean ± SD** | **Adjusted *P* value** | **Significance** |
| --- | --- | --- | --- | --- |
| 28°C | *CEA17Δku80* | 29.83 ± 1.61 | — | — |
|  | *Δshc1* | 29.90 ± 2.01 | 0.9998 | ns |
|  | *Δshc2* | 36.70 ± 0.26 | 0.0005 | *** |
|  | *Δshc3* | 31.23 ± 0.25 | 0.4536 | ns |
| 37°C | *CEA17Δku80* | 60.60 ± 0.35 | — | — |
|  | *Δshc1* | 62.43 ± 0.60 | 0.0193 | * |
|  | *Δshc2* | 69.97 ± 0.57 | <0.0001 | **** |
|  | *Δshc3* | 61.27 ± 0.90 | 0.4741 | ns |
| 48°C | *CEA17Δku80* | 12.63 ± 0.76 | — | — |
|  | *Δshc1* | 13.20 ± 0.96 | 0.6257 | ns |
|  | *Δshc2* | 12.57 ± 0.21 | 0.9986 | ns |
|  | *Δshc3* | 10.93 ± 0.51 | 0.0369 | * |

Table S7 **Statistical comparison of endpoint OD600 values at 30 h among *CEA17Δku80* and *Δshc* mutants in RPMI-1640 medium.**

| **Medium** | **Strain** | **OD600, mean ± SD** | **Adjusted *P* value** | **Significance** |
| --- | --- | --- | --- | --- |
| RPMI-1640 | *CEA17Δku80* | 0.0901 ± 0.0129 | — | — |
| RPMI-1640 | Δ*shc1* | 0.0796 ± 0.0057 | 0.4236 | ns |
| RPMI-1640 | Δ*shc2* | 0.0855 ± 0.0133 | 0.8825 | ns |
| RPMI-1640 | Δ*shc3* | 0.0819 ± 0.0103 | 0.6013 | ns |

Table S8 **Information on clinically isolated *Aspergillus fumigatus* strains**

| Strains | Country or City | *Cyp51A* mutation | MIC value (μg/mL) | | | Azole |
| --- | --- | --- | --- | --- | --- | --- |
|  |  |  | ITC | VOR | POS |  |
| C94 | Shanghai | TR34/L98H | ≥16 | 2 | 1 | resistant |
| C96 | Shanghai | TR34/L98H/S297T/F495I | ≥16 | 1 | 0.5 |  |
| C116 | Fuzhou | TR34/L98H | ≥16 | 4 | 0.5 |  |
| XJ138 | China | TR34/L98H | ≥16 | 2 | 0.5 |  |
| C1664 | Shanghai | TR34/L98H | ≥16 | 8 | 1 |  |
| STJ0105 | China | TR34/L98H | ≥16 | 8 | 2 |  |
| C79 | Shijiazhuang | D262Y | 0.25 | 0.5 | 0.06 | sensitive |
| C490 | Jinan | N248K | 1 | 0.25-0.5 | 0.125 |  |
| E509 | Changsha | N248K | 0.5-1 | 0.25 | 0.06-0.125 |  |
| E631 | Beijing | None | 0.25 | 0.25-0.5 | 0.125 |  |
| E1069 | Kashi | A9T | 0.25-0.5 | 0.5 | 0.125 |  |
| E1109 | Haikou | None | 0.25 | 0.5 | 0.06 |  |

Figure S1. **Construction and validation of *Δshc* mutants.** (A) Schematic diagram of the gene deletion strategy used to replace the target *shc* open reading frame with the hygromycin resistance cassette (*hph*) through homologous recombination. The positions of primers used for target-gene PCR and 5′/3′ junction PCR are indicated. (B) Diagnostic PCR screening of *Δshc1* transformants. Target-gene PCR was used to examine loss of the *shc1* open reading frame, and 5′/3′ junction PCR was used to verify correct integration of the deletion cassette. Transformant 4 showed the expected PCR pattern and was selected as the verified *Δshc1* mutant for subsequent analyses. (C, D) Diagnostic PCR verification of *Δshc2* (C) and Δshc3 (D) mutants. PCR amplification of *tubulin* was included as a genomic DNA quality control. Target-gene PCR and 5′/3′ junction PCR confirmed replacement of the corresponding *shc* locus by the *hph* cassette. (E–G) RT-qPCR analysis of *shc* homolog expression in *Δshc1* (E), *Δshc2* (F), and Δ*shc3* (G). Relative transcript levels were calculated using *tub1* as the reference gene and normalized to the corresponding transcript level in *CEA17Δku80,* which was set to 1. The dashed line indicates the expression level of *CEA17Δku80*. Data are shown as mean ± SD from three biological replicates. ns, not significant; **, P < 0.01; ****, P < 0.0001.

Figure S2 **Effects of individual *shc* deletion on temperature-dependent expression and stress-related phenotypes in *Aspergillus fumigatus*.** (A) Relative transcript levels of *shc1*, *shc2*, and *shc3* in the *CEA17Δku80* parental strain cultured at different temperatures. Transcript levels were determined by RT-qPCR using *tub1* as the reference gene. For each gene, expression was normalized to its own 37°C group, which was set to 1. (B) Colony growth of *CEA17Δku80* and Δ*shc* mutants under different pH conditions. (C–F) Growth of *CEA17Δku80* and Δ*shc* mutants under stress conditions induced by Congo red (C), CFW (D), farnesol (E), and menadione/menaquinone (F). Data are shown as mean ± SD from at least three biological replicates where applicable. ns, not significant; *, *P* < 0.05; **, *P* < 0.01; ***, *P* < 0.001; ****, *P* < 0.0001.

Figure S3. **Targeted metabolite profiling and PI uptake analysis of *Δshc* mutants.** (A, B) Targeted GC-MS quantification of ergosterol (A) and the hopane-type triterpenoid Hopane-3β,22-diol (B) in *CEA17Δku80* and *Δshc1*. (C) Batch-matched relative abundance of ergosterol and Hopane-3β,22-diol in *Δshc* mutants. Because targeted metabolite profiling was performed in two experimental batches, metabolite abundance in each mutant was normalized to the corresponding batch-matched CEA17Δku80 control, which was set to 100%. (D) Representative bright-field, PI fluorescence, and merged images of *CEA17Δku80* and *Δshc* mutants after treatment with 0.25 μg/mL voriconazole (VOR) for 24 h. Scale bars, 50 μm. (E, F) Quantification of relative PI fluorescence intensity after VOR treatment (E) and under basal conditions (F). For each biological replicate, multiple randomly selected fields were analyzed, and the mean value was used for statistical analysis. PI fluorescence intensity was quantified using ImageJ and normalized to the corresponding *CEA17Δku80* control. Data are shown as mean ± SD. Statistical significance was determined by one-way ANOVA followed by Dunnett’s multiple-comparison test. ns, not significant; *, P < 0.05; **, P < 0.01; ****, P < 0.0001.
